# Skin Penetration and Permeation Properties of Transcutol®—Neat or Diluted Mixtures

**DOI:** 10.1208/s12249-018-1196-8

**Published:** 2018-11-12

**Authors:** David W. Osborne, Jasmine Musakhanian

**Affiliations:** 1 Arcutis, Fort Collins, Colorado USA; 2 Pharmaceutical Division, Gattefossé North America, Paramus, New Jersey 07652 USA

**Keywords:** Transcutol, skin, penetration, permeation, mechanism

## Abstract

A heightened interest in (trans)dermal delivery is in part driven by the need to improve the existing skin therapies and also the demand for alternative routes of administration, notably for pharmaceutical actives with undesirable oral absorption characteristics. The premise of delivering difficult actives to the skin or *via* the skin however is weighed down by the barrier function properties of the stratum corneum. Short of disrupting the skin by physical means, scientists have resorted to formulation with excipients known to enhance the skin penetration and permeation of drugs. A vehicle that has emerged over the years as a safe solubilizer and enhancer for a broad range of drug actives is the highly purified NF/EP grade of diethylene glycol monoethyl ether (DEGEE) commercially known as Transcutol®. Whereas numerous studies affirm its enhancing effect on drug solubilization, percutaneous absorption rate, and/or drug retention in the skin, there are few publications that unite the body of the published literature in describing the precise role and mechanisms of action for Transcutol®. In view of the current mechanistic understanding of skin barrier properties, this paper takes on a retrospective review of the published works and critically evaluates the data for potential misses due to experimental variables such as formulation design, skin model, skin hydration levels, and drug properties. The goal of this review is to mitigate the incongruence of the published works and to construct a unified, comprehensive understanding of how Transcutol® influences skin penetration and permeation.

**Graphical Abstract:**
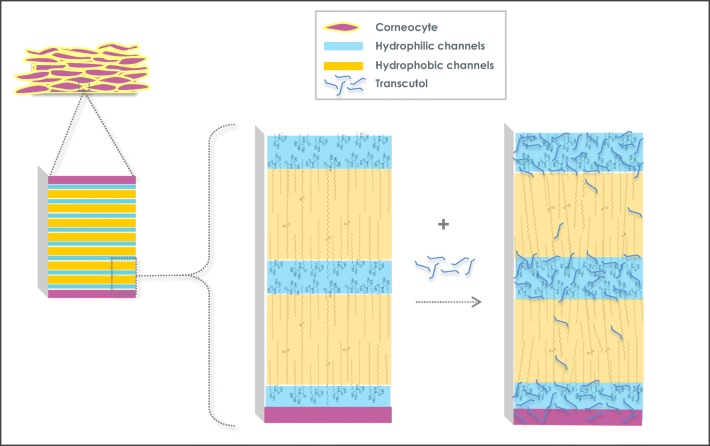
Transcutol has affinity for the hydrophilic head groups of the stratum corneum structures

Transcutol has affinity for the hydrophilic head groups of the stratum corneum structures

## INTRODUCTION

A heightened interest in dermal penetration and permeation enhancement relates to the need for effective new therapies for local as well as systemic delivery. The interest is also propelled by the poor or erratic oral absorption, presystemic elimination, serious side effects, and/or frequent dosing associated with the vast majority of active pharmaceutical ingredients. As alternative route of administration, the skin provides an opportunity for mitigating the aforesaid issues.

The premise of delivering difficult actives by the dermal route however is weighed down by the formidable barrier function properties of the stratum corneum. Short of physically disrupting the skin by physical means (*e.g.*, microneedles), scientists may use formulation approaches that optimize drug/vehicle properties and enable the drug’s penetration and subsequent crossing of the stratum corneum. Designing prodrugs, increasing thermodynamic driving force, ion-pairing to decrease drug charge, eutectic blends, complexation, and complex formulations (such as liposomes, vesicles, nanoparticles) are examples of formulation approaches that have shown to improve the dermal/transdermal delivery of drugs.

A widely practiced formulation option is to use a specific vehicle consisting of an “enhancer” in which the drug is dissolved or dispersed for the purpose of reducing skin’s barrier properties and/or enhancing the quantity/rate of the drug transfer across the stratum corneum. Ideally, the selected enhancer will, alone or in combination with other formulation components, reduce the barrier function of the stratum corneum; facilitate the solubilization of the active in the stratum corneum; and contribute to the active’s subsequent partitioning and diffusion through the stratum corneum. Skin hydration, intercellular lipid disorganization and/or disruption, and restructuring of intracellular keratin are among the mechanisms by which enhancers could exert their role, ideally with minimal lasting impact on the integrity of the skin. A long list of excipients has emerged from decades of scientific search for the ideal vehicle (1–7). Examples of chemical enhancers by functional category are provided in Table I. The list includes alcohols, fatty acid esters, and emulsifiers (some with significant irritation potential) and even harmful substances like terpenes that could extract the skin lipids or dimethyl sulfoxide (DMSO) that induces a garlic-like taste in the mouth after contact with the skin.

**Table I:** Examples of Chemical Enhancers by Functional Category and Chemical Classification (4,6)

Category	Chemical classification	Examples
Hydroalcoholic solvents	Alcohols	Ethanol, isopropyl alcohol, octyl dodecanol, oleyl alcohol
	Ether alcohol	Diethylene glycol monoethyl ether (Transcutol®)
	Glycols	Dipropylene glycol, propylene glycol, 1,2-butylene glycol
Lipophilic solvents	Esters	Ethyl oleate, glyceryl monooleate, glyceryl monocaprate, isopropyl palmitate, isopropyl myristate, propylene glycol monolaurate, propylene glycol monocaprylate
	Fatty acids	Caprylate, caprate, laurate, linoleate, oleate, palmitate, stearate, isostearate
Surfactants	Non-ionic	Brij®, Labrasol®, Labrafil®, Tween® 80
	Anionic	Sodium lauryl sulfate
	Cationic	Alkyl ammonium halides
Others	Misc.	Amides (azone), terpenes, sulfoxides

Among the solvents, a vehicle that has emerged over the years as a safe solubilizer and enhancer for a broad range of drug actives is the highly purified form of diethylene glycol monoethyl ether (DEGEE), commercially known as Transcutol®. Falling in the category of hydroalcoholic solvents (Tables I and II), this excipient is currently referenced in human and veterinary drug products for various routes of administration, notably in dermal and transdermal delivery. In the USA, Transcutol® has been formulated at concentrations of up to 49.9% in topically applied FDA-approved products. This excipient offers advantages over other enhancers for it is clear (transparent) non-volatile and nearly odorless. Unlike oleic acid, azone, and DMSO, Transcutol® does not compromise the integrity of the skin structures. With superior solubilization power, unique physicochemical properties, and well-established safety profile, it is an ideal penetration/permeation enhancer. Nonetheless, its full potential in dermal delivery may be untapped if its role is not thoroughly understood.

**Table II:**
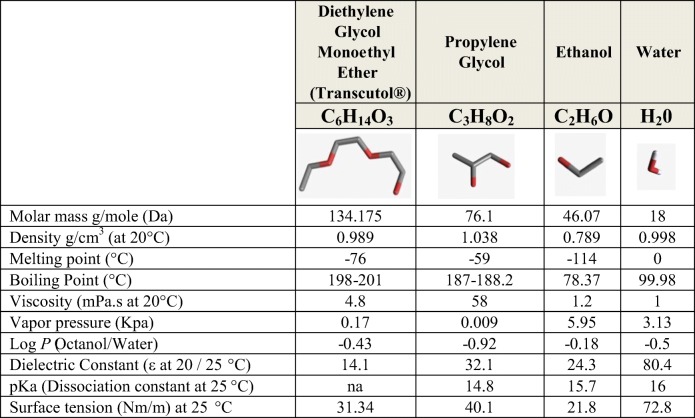
Transcutol Differences and Similarities to Comparable Solvents. Handbook of Pharmaceutical Excipients. Product Data Sheet (8); PubChem (9)

*na* not applicable

Transcutol® can readily penetrate the stratum corneum and strongly interact with the water of the intercellular path to modify the skin permeation of the active pharmaceutical ingredient. Studies involving over 100 different actives affirm its enhancing effect on drug solubilization, percutaneous absorption rate, and/or drug retention in the skin, and yet there are few publications that unite the body of the published literature in describing the precise role and mechanisms of action for Transcutol®. The incongruence of the reports makes it difficult for the drug development scientist to discern the underlying mechanisms and to assimilate the critical knowledge into his/her own work. The goal of this review is to construct a unified, comprehensive understanding of how Transcutol® influences skin penetration and permeation. Following a review of product characteristics, this paper critically evaluates the published data using current mechanistic understanding of skin barrier properties. In the interest of brevity, this review is limited to simple (neat) and diluted systems with Transcutol®.

## PRODUCT ATTRIBUTES

### Identity, Purity, Safety, and Regulatory Aspects

Transcutol® is a highly purified form of ethyl alcohol, known as diethylene glycol monoethyl ether (DEGEE) under EP and USP/NF monographs. Not to be confounded with ethylene glycol ethyl ether (EGEE) or diethylene glycol methyl ether (DGME), Transcutol® has a fully established safety profile. The nonclinical data along with a long history of use as vehicle and solvent by various routes of administration provide evidence for its safety (10). The reader may consult in-depth reviews of the topic by a number of authors, including (8,10–12). The pharmaceutical grades are guaranteed by the manufacturer (8) to have purities of > 99.90% (HP grade) and > 99.80% (P grade). In this manuscript, the P and HP grades are referred to simply as Transcutol (TRC).

Currently, the Inactive Ingredients Database of the US Food and Drug Administration (FDA) lists DEGEE for topical (up to 49.9%) and transdermal (up to 5%) routes of administration. Based on the safety data available, and factoring in a 100 times safety ratio, the manufacturer (8) recommends a limit of 20 mg/kg by the dermal route of administration and 10 mg/kg by the oral route (10). There are no limits set for inactive ingredients’ use level in over-the-counter products, so long as the usage complies with Good Manufacturing Practices in the USA. In Japan, Transcutol is permitted for use in quasi-drug products including personal care (hair, skin) and oral preparations. In Europe, Middle East, and Africa (EMEA) countries, the published guidelines state no use limits for Transcutol in pharmaceutical products. In Australia, therapeutic goods are divided into “medicines” and “medical devices” where Transcutol is approved as active ingredient for prescription medicines, as excipient in devices, and may be used in listed medicines (OTC and prescription) for export purposes. Application of Transcutol as solubilizer and/or penetration/permeation enhancer in pharmaceutical preparations includes spray-on solutions, patches, emulsions, gels, and ointments (13).

### Physicochemical Properties

Transcutol is a clear (transparent) liquid with very low viscosity and characteristic pleasant odor. Its physical and chemical properties are comparable to protic solvents like ethanol (EtOH), propylene glycol (PG), and water. The viscosity, density, and Log *P* properties of Transcutol approach those of water but the vapor pressure and boiling point accost those of PG (Table II). Other properties like surface tension and melting point of Transcutol fall somewhere between those of EtOH and water. The relatively low vapor pressure and high boiling point of Transcutol are advantages over EtOH and water which cannot be processed at high temperatures. Transcutol is chemically stable in neutral and alkali pH, with optimal formulation range of pH 4 to 9. The pH of a 10% Transcutol solution is near neutral (6.6).

An important property of Transcutol is its capacity to dissolve a broad range of hydrophilic and lipophilic actives. Its ability to outperform PG and EtOH in solubilization power makes it a highly useful pharmaceutical excipient (14). With a negative log *P* of ~ 0.5, Transcutol is considered as a polar protic solubilizer that demonstrates affinity and good miscibility with also hydrophobic groups. The ability of solvents having a negative log *P* to readily penetrate the stratum corneum contrasts with lipophilic actives (log *P* values of 2–3) more readily penetrating the stratum corneum than actives having negative log *P* values. Note that all the solvents listed in Table II have negative log *P* values. Dimethyl sulfoxide, generally regarded as the most skin permeable solvent, has a log *P* value of − 1.35. Thus, the expectations for skin penetration for pharmaceutical actives characterized by molecular weights of over 200 and functional groups that carry charge are not necessarily applicable to solvents that cannot be ionized and have molecular weights around 100.

**Fig. 1:**
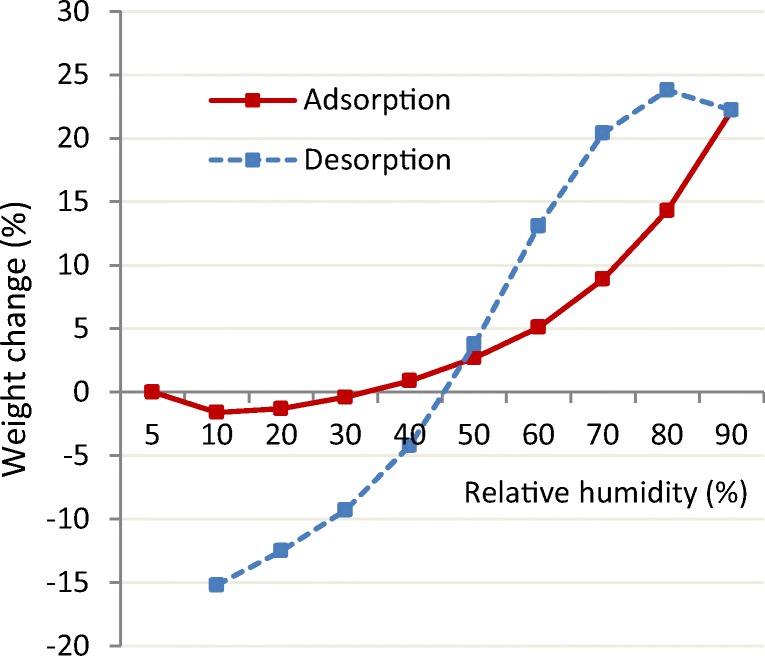
Water sorption/desorption curve of Transcutol (8)

Transcutol is compatible with most pharmaceutical excipients; soluble in common solvents like glycerin, EtOH, PG, and water; miscible with polar lipids like medium-chain triglycerides (MCT) and polyethylene glycol (PEG) based surfactants (polyoxylglycerides); but insoluble in non-polar mineral oil or dimethicone. Owing to its high solubility and miscibility with water, Transcutol may hydrate depending on the relative humidity conditions (RH). The hydration kinetics of Transcutol were observed by Ganem-Quintanar (15) who reported a moisture gain of 6% at 98% RH and a moisture loss of ~ 1% at 50% RH conditions. A recently obtained water sorption/desorption isotherm (Fig. 1) confirms a similar trend.

### Skin Penetration and Permeation of Neat Transcutol

**Fig. 2:**
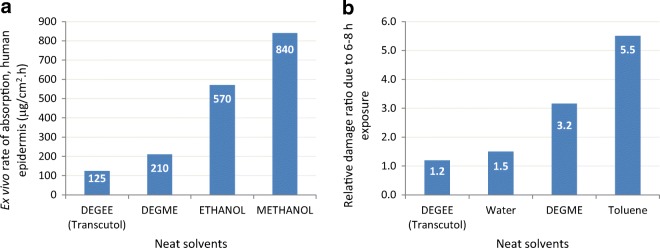
**a** Absorption rate of glycol ethers and alcohols. **b** Relative effect of neat solvents on the skin after 6–8 h of exposure. DEGEE, diethylene glycol monoethyl ether; DEGME, diethylene glycol methyl ether. Adapted from Dugard (16)

Among the many publications reviewed, only a few have focused on the skin absorption properties of Transcutol as a permeant, independent of an active drug (16–18). In an early *ex vivo* study, permeation of neat Transcutol across human abdominal (whole) skin was compared to those of tritiated water, toluene, and other glycol ethers (16). The total amount of Transcutol penetrated over 8 h translated to an absorption rate of 125 ± 103 μg/cm^2^ h with a lag time of < 1 h. Fig. 2a compares the flux of Transcutol with other neat solvents, including ethanol and methanol. The integrity of epidermal membranes was also established before and after contact with the tested vehicles relative to water. The Transcutol permeability constant was 1.32 × 10^4^ cm/h, and the damage ratio (a measure of integrity of the skin) recorded for Transcutol was similar to the values of 1–2 recorded for water (Fig. 2b).

Ganem-Quintanar (15) evaluated the trans-epidermal permeation of Transcutol using whole and stripped hairless rat skin in a modified vertical Franz diffusion cell over 48 h. Per this publication also, Transcutol diffusion is primarily hindered by the stratum corneum, and within 2 h, a constant flux of 580 μg/cm^2^ h in the rat skin is established. Note that this permeation rate is 4.6 times higher than the 125 μg/cm^2^ h reported for the *ex vivo* human skin by Dugard (16) and corroborates with the expected 4 to 8 times higher porosity of the rat *vs.* human skin models reported by Ritschel (19). Ganem-Quintanar (15) concluded that the penetration enhancing effect of Transcutol “may be attributed, on one hand, to its permeation into the skin and its effect on the partition behavior of a drug, and on the other hand, to the hydration of Transcutol which can absorb water not only from the air, but also from the skin (change of vehicle composition)”, thereby maximizing the drug thermodynamic activity due to a change of drug solubility.

**Fig. 3:**
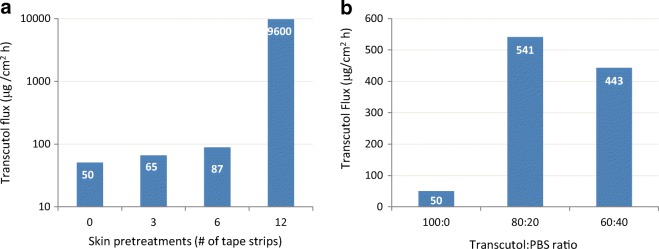
**a** Transcutol flux across non-stripped and stripped (3, 6, and 12 times) skin samples of 5-day-old rat. **b** Transcutol flux as a function of dilution with phosphate buffer. Adapted from Koprda (17)

The work of Koprda (17) showed that the skin resistance to the permeation of neat Transcutol is fully determined by the stratum corneum barrier. The permeation properties of radiolabeled Transcutol through whole and tape-stripped abdominal skin of 5-day old rats were evaluated using Franz diffusion cells. Fig. 3a illustrates the results for neat Transcutol permeating at 50 μg/cm^2^ h across the untreated (non-stripped) skin and increasing to 87 μg/cm^2^ h after 6 strippings; in the samples pre-stripped 12 times, however, the skin barrier function had disappeared resulting in a 200-fold increase in the Transcutol flux, to ~ 10 mg/cm^2^ h. Fig. 3b demonstrates that the flux of Transcutol diluted with phosphate buffer (PBS) in 60:40 and 80:20 ratios was significantly higher (9- to 10-fold) than that observed for neat Transcutol. As for the retention of Transcutol in the skin, only 5.3% of the applied Transcutol was detectable in the tested tissue, of which 70% (3.5% of total Transcutol dose) was detected in the top 10 strips.

**Fig. 4:**
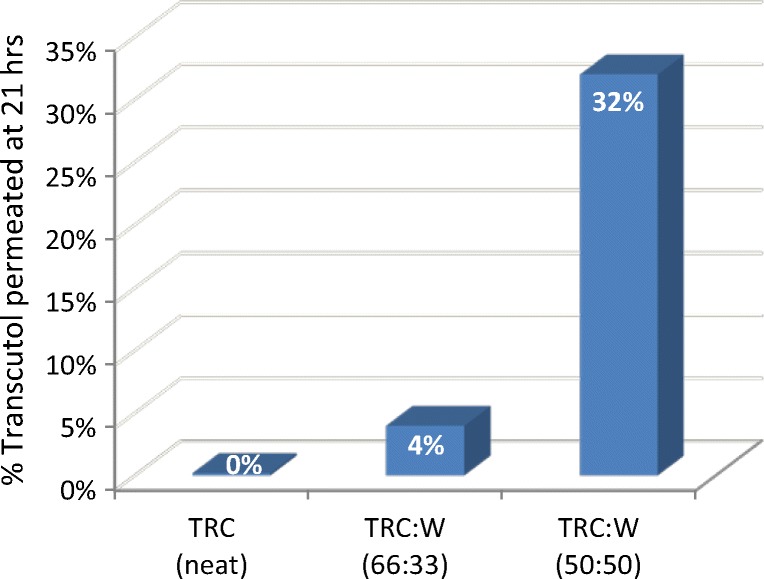
Permeation of neat and diluted Transcutol across whole rat skin. Koprda (18)

A later publication by Koprda (18) revealed even a larger and statistically significant positive effect of water (W) on the permeation of Transcutol, notably at 1:1 (TRC/W) ratio (Fig. 4). These findings (17,18) can be explained by the high affinity of Transcutol for water and corroborate with much of the literature published in the decades that have followed since.

**Fig. 5:**
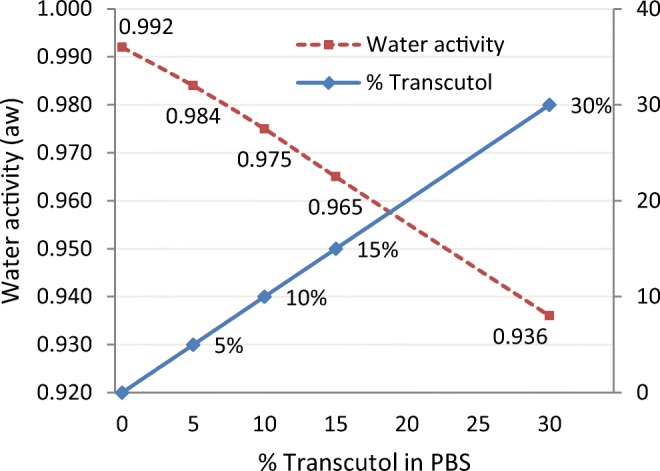
Relationship between Transcutol concentration in phosphate buffer (PBS) and water activity in the donor solution. Adapted from Bjorklund (22)

The importance of water and conversely its absence from Transcutol formulations in IVPT studies (15,17,18,20,21) becomes apparent in view of the work published by Bjorklund (22). Combining impedance spectroscopy with polarization transfer solid-state NMR techniques, the authors demonstrated that increased hydration leads to mobilization of the non-aqueous stratum corneum components, increased solubility, and reduced diffusional resistance toward permeants (drugs, excipients) in a formulation. In this study (22), the flow-through cell was loaded with hydrated dermatomed pig skin (approximately 0.5 mm), dosed at 3145 mg/cm^2^ for 24 h. The flux of Transcutol across hydrated pig ear skin increased with increasing concentration of Transcutol in the donor solution. At steady state, the flux of the 30% Transcutol in PBS (231 ± 40 μg/cm^2^ h) was ~ 4 times higher than that obtained with a 5% Transcutol in PBS (55 ± 10 μg/cm^2^ h). The molecular mobility of the stratum corneum protein or lipid components in the presence of Transcutol resembled those of PBS soaked skin more than the molecular rigid stratum corneum at 93% RH. The study also found that increasing concentrations of Transcutol (up to 30%) lowered the water activity of the stratum corneum (Fig. 5), imposing dehydrating conditions while retaining the high skin permeability of a hydrated stratum corneum at reduced hydration conditions. The authors added that increased mobilization due to hydration relates primarily to reversible processes that facilitate the movement of excipients and/or actives through the intercellular route. Monitoring and controlling such changes may be used as tools for regulating drug transport across the skin (22).

Ganem-Quintanar (15) had previously shown that application of neat Transcutol in IVPT experiments increased outflow of water from the receptor solution into the donor compartment of the Franz cell and changed the composition of the donor solution. When 1 ml (1000 μg) of neat Transcutol was applied to the donor compartment of a Franz cell (2.54 cm^2^) loaded with whole rat skin (0.9% saline as the receptor solution) significant “back flux” of water from the receptor compartment into the donor compartment occurred. After a 48-h experiment, the donor compartment no longer contained neat Transcutol, but rather consisted of a 15:85 W/TRC blend. This corresponded to a water “back flux” value of 1820 μg/cm^2^ h. Although this “back flux” of water undoubtedly hindered Transcutol transport from the donor phase to the receptor solution, Transcutol passed through whole rat skin at a constant rate of 580 μg/cm^2^ h over a 32-h dosing duration. The authors remarked that “these two opposite fluxes could affect the permeation rate of a given drug by changing its partition into the skin as well as its thermodynamic activity in the donor solution.”

The dependence of Transcutol permeation on dilution with water or PBS can be explained in part by the high affinity of Transcutol for water. In the absence of water, neat Transcutol is likely attracted to the aqueous regions of the bilayers in the stratum corneum, becoming immobilized (retained), and in certain instances increase the stratum corneum rigidity and reduce its permeability. In aqueous formulations on the other hand, Transcutol is likely trailing the abundance of water, thus permeating at a faster rate. Effect of skin hydration dynamics and skin retention of Transcutol is further discussed in the “Drug Penetration and Permeation Mechanisms” and “Discussion” sections.

In summary, the permeation rate of Transcutol is significantly influenced by the stratum corneum, the skin model, the level of hydration of the skin, and the presence of water in the donor (formulation). The different animal species used as a skin source to generate these IVPT results undoubtedly contribute to the wide range of Transcutol flux values. Nevertheless, some generalizations may be made: A flux of 580 μg/cm^2^ h for rat skin (15) compares to 125 μg/cm^2^ h in human skin (16) and 0.87 μg/cm^2^ h for bovine skin (21).

## DRUG PENETRATION AND PERMEATION MECHANISMS

A number of excellent reviews (1–7) discuss commonly used penetration enhancers and their properties. It is widely understood that skin penetration enhancement can occur by solvents that participate in the optimization of drug and vehicle properties and/or modification of the stratum corneum homeostasis (23). These two conditional pathways often coexist and in the case of Transcutol intercalate, further complicating the interpretation of data produced by conventional diffusion models of *in vitro* permeation testing (IVPT). Fortunately, current investigative techniques such as Fourier transform infrared (FTIR), wide- and small-angle X-ray diffraction, and differential scanning calorimetry (DSC), when used alongside the IVPT data have helped differentiate the underlying mechanisms of skin penetration and permeation with Transcutol.

A critical evaluation of the published results points to key mechanisms whereby Transcutol may exert a role in the *optimization of drug/vehicle properties*, namely (i) increasing thermodynamic driving force and (ii) decreasing drug charge. Transcutol may also have an effect on the *modification of the stratum corneum*, specifically (i) increasing drug solubility/partitioning in the stratum corneum; (ii) maintaining hydrated dynamics in the stratum corneum; and (iii) intercellular lipid fluidization. These minimally referenced mechanistic narratives are supported below by a review of the literature that details IVPT parameters, lists permeation results, and provides calculated enhancement ratios for drugs to facilitate comparison between publications, followed by a broader examination of published works in the “Discussion” section. This review focuses mainly on the mechanisms associated with neat, diluted, and simple blends of Transcutol with other enhancers in simple solutions, suspensions, and gels of non-penetrating, non-occlusive polymers.

### Optimization of Drug and Vehicle Properties

#### Enhancing Skin Penetration by Increasing Thermodynamic Driving Force

The concept of maximum thermodynamic driving force occurring at saturation is based on Fick’s laws of diffusion that describes the formation of a concentration gradient from a region of high concentration (donor phase in a skin diffusion cell) to a region of lower concentration (sink conditions in the receptor phase in a skin diffusion cell) (24). For Fick’s laws to rigorously hold, several conditions must be met including (1) the donor solution is inert with regard to the permeability of the membrane and (2) that a concentration gradient does not develop within the donor solution (usually satisfied by the donor solution being applied as an infinite dose). At saturation, the driving force is unity, and since supersaturation can raise the driving force to significantly above unity, formulating at the same fraction of active solubility (say 1% of saturation) in two different donor solutions will provide the same thermodynamic drug activity.

**Fig. 6:**
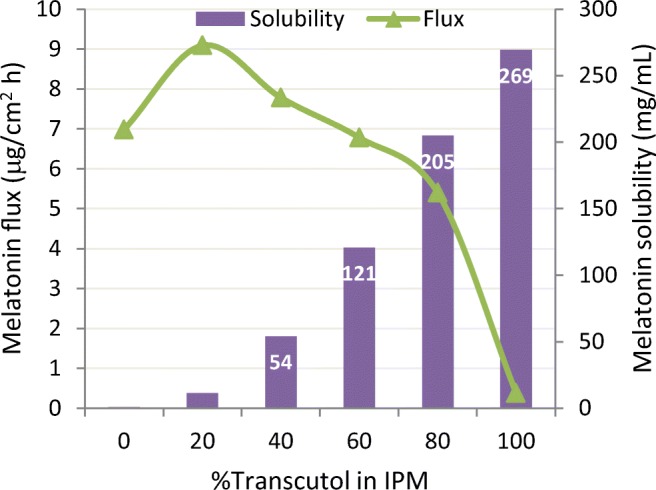
Melatonin flux and solubility in varying combinations of Transcutol and isopropyl myristate. Adapted from Gwak (25)

Transcutol, a solvent that can increase drug solubility by orders of magnitude compared to other topically applied pharmaceutical solvents, can have a dramatic influence on thermodynamic driving force. If thermodynamic activity is not considered, it may appear that Transcutol retards rather than enhances skin penetration. For example, just looking at Fig. 6 derived from the skin flux values reported by Gwak (25) and ignoring thermodynamic activity, one could presume that isopropyl myristate (IPM) enhances skin penetration 10-fold while Transcutol reduces to half the ability of melatonin to cross the skin. However, a closer examination of the permeation rate *vs.* solubility and comparing drug concentration, flux (*J*_S_ in μg/cm^2^ h), lag time (*T*_L_) and solubility values reported in the same study (Table III) reveals a different picture. Since the Transcutol solution was dosed at 3.7% of its saturated thermodynamic activity compared to buffer or IPM dosed at 100% of the thermodynamic activity, conclusions concerning the ability of Transcutol to either enhance or retard skin penetration cannot be made based on the data set presented. Infinite dose *in vitro* skin permeation studies are usually completed at equivalent thermodynamic driving force to allow comparison of data from different donor solutions. Any IVPT experiment that doses at equal concentrations of active, which is typical at later stages of a commercial development program, should consider if an additive is significantly changing solubility of the active, *i.e.*, significantly changing thermodynamic driving force.

**Table III:** Melatonin Permeation Properties from Three Vehicles Having Different Drug Solubility and Saturation Limits. From Gwak (25)

Vehicle	Drug concentration	*J*_S_ (μg/cm^2^ h)	*T*_L_ (h)	Solubility (mg/ml)
PBS (pH 6.02)	Saturated	0.66 ± 0.10	4.15 ± 2.57	1.12 ± 0.08
Transcutol	10 mg/ml	0.38 ± 0.05	2.86 ± 0.31	269.2 ± 11.4
IPM	Saturated	6.98 ± 4.04	2.58 ± 0.85	0.71 ± 0.12

**Fig. 7:**
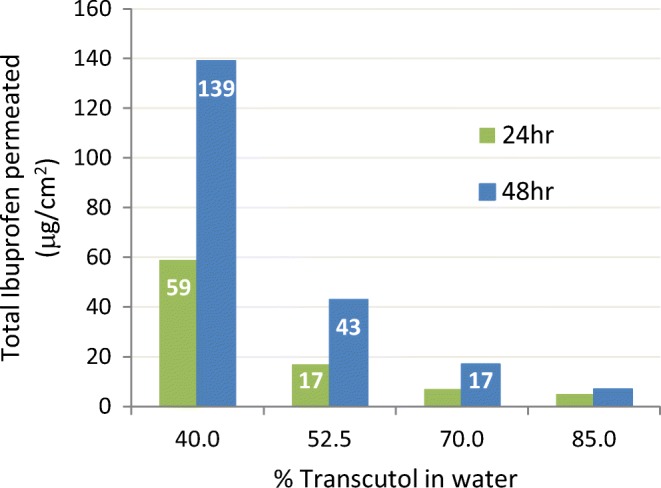
Ibuprofen (5% *w*/*w*) permeation across human female skin as a function of Transcutol/water binary systems. From Bialik (20)

Demonstrative of the thermodynamic driving force at play is an IVPT work by Bialik (20). The study reported that ibuprofen, when fully dissolved in Transcutol, did not produce any measurable flux across human skin. However successive dilutions with water to form binary TRC/W mixtures (Fig. 7) resulted in an increased permeation of ibuprofen. Ibuprofen solubility in Transcutol (400 mg/ml) is considerably higher than in water (0.021 mg/ml). The authors explained that on addition of water in which ibuprofen is less soluble, an increasing amount of ibuprofen falls out of solution, creating a higher thermodynamic activity which translates to increase of skin permeation rate.

Attention to the concept of thermodynamic driving force is of paramount importance when designing an IVPT study to avoid confounding the data. From a practical product development point of view, a formulator never develops a topical product at saturation due to the likelihood that the active will precipitate during temperature excursions. Also, when clinically relevant doses of a topical product are applied to the patient, drug product metamorphosis immediately alters the thermodynamic driving force, so other considerations become of equal or greater importance during development of dermatological products. Gordon Flynn (26) provided an excellent conceptual review of thermodynamic driving force as related to topical products compared to transdermal skin delivery systems.

#### Enhancing Skin Penetration by Decreasing Drug Charge

The ionization equilibrium of a drug is affected by adding a solvent like EtOH or Transcutol to an aqueous solution. Known as the solvent effect, anytime a liquid having a dielectric constant lower than the dielectric constant of water (*ɛ* = 78) is added to an aqueous solution of ionized drug, the solvent can preferentially solvate the drug and prevent it from completely ionizing by forming ion pairs in the low dielectric medium. This decrease in drug charge by addition of a low dielectric solvent may significantly increase the amount of unionized drug partitioning into and permeating through the skin. Maitani (27) showed that EtOH increased the amount of unionized sodium diclofenac by encouraging ion pair formation in the low dielectric constant formulation and significantly increased skin penetration. Modifying a formulation to reduce drug charge by encouraging ion pair formation is a well-established technique of enhancing skin penetration (23).

The recognition that solvents such as ethanol (*ɛ* = 24) and Transcutol (*ɛ* = 13) may be increasing skin penetration of certain actives by reducing charge has rarely been discussed in the IVPT literature. Within the “Discussion” section, we provide a critical review of publications involving charged/ionizable drugs.

### Modifying the Stratum Corneum

#### Increasing Drug Solubility in the Stratum Corneum

Transcutol has been shown to alter drug penetration and permeation of the skin by diffusing into the stratum corneum and altering the solubility parameter of the intercellular lipid domain (28). A 1936 thermodynamic description of solubility by Hildebrand, later refined by Hansen (29) assigned a numerical value called the solubility parameter, to each solvent and solute based upon their cohesive energy density. The solubility parameter of the skin is influenced by the relatively high hydrogen bonding of the water molecules and the tight packing of the intercellular lipids in the stratum corneum. The energy required to separate a permeant (drug) from its solvent (enhancer), *i.e.*, partitioning of the drug into the stratum corneum may be facilitated by a differential in solubility parameters between skin, drug, and enhancer (3,30). More specifically, low molecular weight solvents such as Transcutol can readily permeate the intercellular spaces and lower the solubility parameter of the intercellular lipid domains to better match the solubility parameter value typical of pharmaceutical actives. This in turn increases the solubility of the permeant (active) in the stratum corneum. Others (30–33) have shown that Transcutol enhances a permeant’s solubility in the skin without significantly influencing the diffusivity of the permeant in the skin/stratum corneum Osborne (14). Using electron microscopy, Ritschel (34) showed that Transcutol is able to significantly contribute to the swelling of the intercellular spaces of the stratum corneum. This unique ability to swell the intercellular path of the skin’s barrier gives rise to both skin retention (the intracutaneous skin depot) and skin penetration enhancement, which may seem mechanistically contradictory unless the molecular structure of the active is taken into consideration.

Keep in mind that the phrase “altering the solubility parameter of the intercellular lipid domain” is just one way of articulating that Transcutol, while present in the stratum corneum, dramatically changes how a drug will partition into the skin from the topical product residing on the skin surface. As described, in an infinite dose, steady-state flux IVPT experiment, Transcutol promptly partitions into the stratum corneum and saturates the intercellular lipid domain. This allows greater amounts of drug to partition into the stratum corneum. The ability of the drug to partition out of the stratum corneum and into the viable epidermis is dependent upon the physical chemical properties, *i.e.*, molecular structure, of the drug. If the drug readily partitions out of the stratum corneum, then increased partitioning into the skin due to the presence of Transcutol results in increased skin permeation. Drugs that do not carry a charge at the physiological pH value of the epidermis and have an octanol-water partition coefficient above 3.0 are likely to have decreased partitioning out of the stratum corneum. For these actives an enhanced penetration due to the presence of Transcutol results in increased concentration and retention of the drug in the stratum corneum. As expressed, a topically applied solvent that increases drug solubility in the stratum corneum will be a skin penetration enhancer only if the active readily partitions out of the stratum corneum due to good solubility of the active in the viable epidermis. This description is most relevant to IVPT studies applying an infinite dose of the test article. When applying a clinically relevant dose that can undergo drug product metamorphosis, this description is often an oversimplification. Note that this conceptual description also ignores any penetration that might occur by a shunt pathway, *i.e.*, the pilosebaceous unit. In general, the large enhancement ratios published from infinite dose single application *in vitro* studies rarely translate into multi-fold enhancement when finite doses of the same product are administered in the clinic over a full treatment cycle. However, the ranking of “most bioavailable” formulation to “least bioavailable” formulation usually holds for IVPT compared to clinical results.

#### Enhancing Penetration by Maintaining Hydrated Dynamics in the Stratum Corneum

Hydration of the skin can enhance skin penetration for both hydrophilic and lipophilic permeants. Extensive hydration is shown to increase water binding to keratin in the stratum corneum cells (1,19,35), thereby swelling the corneocytes and loosening the intercellular lipid packing. The effect is a significant decrease of its diffusional resistance (increased drug diffusivity) in the lipid pathway (36–38). It is important to emphasize that water is constantly back diffusing from the viable epidermis out through the stratum corneum to evaporate in the air (38). This phenomenon is labeled as insensible perspiration or trans-epidermal water loss (TEWL). Depending on levels of ambient humidity, at equilibrium, the water content of the stratum corneum is normally around 15–20% of the dried weight of this outermost layer of the skin. Any formulation component or physical barrier, *i.e.*, occlusion, that delays evaporation and results in increased skin hydration can potentially increase skin penetration of a topically applied drug.

It was shown by Bjorklund (22) that application of Transcutol to the skin can be used to retain the high skin permeability of a hydrated stratum corneum at reduced hydration conditions. Once Transcutol permeates the stratum corneum, the molecular mobility of the stratum corneum protein or lipid components is more similar to PBS soaked skin than the molecular rigid stratum corneum at 93% RH. It was further proposed by Bjorklund that Transcutol has similar physicochemical properties as urea, glycerol, or other natural moisturizing factors and may influence molecular dynamics of the stratum corneum in a similar manner. Although we think of Transcutol as an excellent solvent for lipids, it must be noted that Transcutol is uniquely hygroscopic.

It is therefore postulated that the ability of Transcutol to readily penetrate the stratum corneum and strongly interact with the water of the intercellular path is what makes Transcutol unique when compared to other solvents that modify skin permeation. The effects of water and other solvents on drug penetration and permeation from Transcutol formulations are further discussed in the “Discussion” section.

#### Enhancing Skin Penetration by Modifying Epidermal Lipid Structure

Skin penetration enhancers such as azone and oleic acid (OA) have been shown to increase permeability by significantly disordering (fluidizing) the bilayered lipid structures of the intercellular domain of the stratum corneum. Lipid disruption can be shown by DSC to measure the phase transition temperature of the intercellular lipids or by FTIR spectroscopy to measure rotational bands linked to the intercellular lipids. Earlier publications utilizing these techniques to study Transcutol (25,28,39–41) did not find Transcutol alone to disrupt the epidermal lipid structure or the structural integrity of the skin. In a comparative study of a dozen solubilizers and penetration enhancing excipients including fatty acids, Gwak (25) concluded that Transcutol merely eases the partition of the active by increasing its solubility in the skin which then leads to improved drug partitioning in the skin. Salimi (42) using DSC and FTIR evaluated the effectiveness of different vehicles on meloxicam permeability and microstructure of intercellular lipids in the stratum corneum layer of skin. The results showed drug diffusion to be the rate-limiting step for the permeability (flux) of meloxicam into the full rat skin. Among the enhancers studied, Transcutol was the most effective, increasing flux 21 times and diffusion coefficient 17 times compared to hydrated skin. The principal mode of action of Transcutol enhancement activity was attributed to the facilitation of drug partitioning and diffusion into skin. A thorough mechanism of percutaneous penetration enhancement study of Transcutol was published by Harrison (28). Using 4-cyanophenol as the skin permeant, the diffusivity and solubility of this molecule in human stratum corneum was followed using attenuated total reflectance Fourier transform infrared (ATR-FTIR) spectroscopy and Franz-type diffusion cell flux measurements of the permeant. These methodologies were performed on skin dosed with a mixture of Transcutol and water (1:1 ratio) saturated with 4-cyanophenol. The 50:50 TRC/W blend increased the flux of 4-cyanophenol across human skin *in vitro* by a factor of approximately two compared to a saturated water control. The authors concluded that ATR-FTIR spectroscopy indicates that Transcutol does not appear to have a fluidizing effect on structured lipids, but rather enhances skin penetration by increasing the solubility of the penetrant in stratum corneum.

Generally, disturbance of the crystalline packed lamellar structures of the stratum corneum lipids, *e.g.*, changes in the their natural orthorhombic arrangement to a hexagonal or liquid phase may help improve the permeability of molecules (43). However, a relatively small number of publications have studied the impact of Transcutol on this region of the stratum corneum. Applying small- and wide-angle X-ray scattering techniques, Moghadam (43) examined the effect of various penetration enhancers on the structural changes and hydrocarbon chain packing of the human stratum corneum lipids. As expected, the highest degree of stratum corneum lipid disordering was caused by surfactants and terpenes. The skin samples pretreated with neat solvents (EtOH and PG) were unaffected. As for Transcutol, it was not tested neat nor diluted with water, but rather at 10% in PG for which a slight disordering effect within the stratum corneum was reported. The authors suggested an increase in membrane fluidity due to Transcutol without elaborating on the potential additive impact of a TRC/PG blend on the stratum corneum structures. The effect of adding solvents to Transcutol are discussed in the “Effect of Transcutol Binary Solvent Blends on Drug Penetration/Permeation” section.

Combining polarization transfer solid-state NMR methods and impedance spectroscopy measurements, however, Bjorklund (22) found that Transcutol slightly increases both the mobility of the keratin filament terminals and stratum corneum lipids. The described effect was limited to the relatively polar regions of the stratum corneum, notably the ceramide head groups of lipid lamellae. Increased mobility in the ceramide head groups indicates that Transcutol can preferentially partition into more polar environments and disturb the packing in the interfacial head group layer of intracellular lipids. This slight disturbance was similar in scale to that observed with neat PBS. This implies that Transcutol can disturb the lipid packing by preferentially partitioning in the more polar regions of the stratum corneum.

Other studies have shown that when Transcutol is combined with enhancers known to disrupt epidermal lipid structure such as azone (18,44), significant skin penetration enhancement occurs. It should be noted that fluidization of the intercellular lipid domain should always cause an increase in permeability coefficient and decrease in lag time. Whenever a significant increase in skin flux occurs after a significant increase in lag time, the possibility of lipid extraction into the donor phase and modification of the IVPT parameters must be considered.

## DISCUSSION

### Effect of Neat Transcutol on Drug Penetration and Permeation

Five studies (5,36,45–47) that have characterized drug flux from saturated solutions of neat Transcutol, comparing the results against drug flux from saturated solutions of water. Summarized in Table IV, these studies involved Franz cell experiments using saline as the receptor solution or PBS (pH = 7.4 for lidocaine) except for ondansetron HCl and tenoxicam which were run on side-by-side cells using saline/PEG 400 (60:40) as the receptor. Table IV provides the saturation concentration of the drug (mg/ml) in Transcutol and in water that was dosed, the skin flux values, the enhancement ratio (ER) which is calculated from the drug flux from neat Transcutol divided by the drug flux value obtained from water (control), physical chemical properties of the drug, and details concerning IVPT parameters. Listed in the order of increasing values for flux ER, the results show that the four salts of diclofenac and ondansetron had lower skin flux values when delivered from neat Transcutol compared to water. These publications are discussed below including a concise statement of the conclusions from each study and a critical evaluation of the IVPT methodology.

**Table IV:** Effect of Neat Transcutol on Permeant Penetration and Permeation (5,36,45–47)

Study reference	Active (permeant)			Study conditions		Active saturation concentration (mg/ml)		24 h flux (μg/cm^2^ h)	Enhancement ratio^*a*^
	Description	MW	Log *P*	Membrane	Dose (mg/cm^2^)	Transcutol	Water		
2007 Minghetti (47)	Diclofenac epolamine	411	0.9	Human-SCE^*b*^	1752^*c*^	0.43	0.56	0.03	0.01
	Diclofenac sodium	318	2.94	Human-SCE	1752^*c*^	0.66	0.37	0.06	0.03
	Diclofenac diethylamine	369	0.2	Human-SCE	1752^*c*^	0.28	0.02	0.96	0.17
	Diclofenac potassium	334	1.1	Human-SCE	1752^*c*^	0.71	0.22	0.84	0.62
2004 Gwak (46)	Ondansetron HCl	293	2.4	Hairless mouse	4688	18.1	36	4.3	0.09
1993 Bonina (36)	Caffeine	194	− 0.1	Human-SCE	133	14.1	27.9	0.635	0.88
	Testosterone	288	3.32	Human-SCE	133	104	0.03	0.231	0.88
2014 Bayldon (5)	Lidocaine	234	2.4	Sheep-clipped	88.5^*c*^	0.56	3.8	65.4^*d*^	4
2002 Gwak (45)	Tenoxicam	337	2.2	Hairless mouse	4688	4.74	0.01	1.13	7.5

^*a*^Permeation enhancement ratio is drug flux from saturated Transcutol solution divided by flux from saturated water solutions of the drug

^*b*^SCE = stratum corneum + epidermis

^*c*^μl/cm^2^ being equivalent to mg/cm^2^ if the density of the donor solution were 1.0 mg/ml

^*d*^Flux calculated after 8 h

Minghetti (47) studied four different salts of diclofenac dissolved in four different solvents (W, PG, TRC, and OA), demonstrating that in aqueous solutions, different species of diclofenac exist having different ability to permeate the skin. For each salt studied, the overall permeability was due to the partial contribution of each species, *i.e.*, diclofenac anion, ion pairs, and acidic diclofenac. It was shown that for the saturated water systems, the diethylamine and epolamine salts form ion pairs that have a greater ability to partition into the stratum corneum compared to diclofenac sodium or diclofenac potassium. When dissolved in Transcutol, Minghetti (47) used the solvent effect to explain that Transcutol results in ion pair formation, greater partitioning of the neutralized diclofenac salt into lipid domains of the horny layer, and ultimate dissociation of the ion pairs when diclofenac reaches the aqueous viable epidermis. The researchers did not discuss that flux of the diclofenac salts from Transcutol ranged from 1/100 to 2/3 of the flux values from saturated water. When viewed in light of the Bjorklund (22) study, it would appear that enhanced penetration resulting from a decrease in ionized diclofenac (solvent effect from using neat Transcutol) is overwhelmed by stiffening of the intercellular domain (increased skin barrier) due to stratum corneum dehydration that occurs when dosing with neat Transcutol.

Gwak (46) used IVPT to determine skin flux values and permeability coefficients from saturated solutions of Transcutol and water. Hairless mouse skin was mounted on a side-by-side diffusion cell. As seen in Table IV, ondansetron HCl aqueous saturation concentration of 36 mg/ml was about twice the amount of active found to be soluble in Transcutol. It should be noted that ondansetron is a weak base (pKa = 7.4) that is ionized under acidic conditions. The natural pH of ondansetron HCl solutions is about 4.5 to 4.6 and the solubility is significantly reduced in solutions at, or above, pH equal to 6 (48). Since the calculated (ALOGPS) aqueous solubility of ondansetron HCl is 0.248 mg/ml, it can be assumed that the active in the donor compartment of the side-by-side cell was completely ionized. The Gwak study (46) showed that the flux of ondansetron from neat Transcutol was an order of magnitude lower than from a saturated aqueous solution. Likewise, the apparent permeability coefficient of ondansetron delivered from water was 13.4 × 10^−7^ cm/s compared to 1.21 × 10^−7^ cm/s when delivered from neat Transcutol. Since the saturated solutions are at equivalent driving force, any solvent effect benefit from formulating ondansetron in neat Transcutol is overwhelmed by the stratum corneum dehydrating effect of neat Transcutol. Also, since the solubility is dramatically reduced at pH values above 6, ondansetron will not readily partition out of the stratum corneum into the viable epidermis which is at physiological pH.

Bonina (36) studied two model drugs having vastly different physical properties, caffeine and testosterone. Saturated solutions of caffeine and testosterone from seven neat solvents and a blend of PG and propylene glycol dipelargonate (DPPG) were applied to human skin loaded onto Franz cells. The flux values for both model drugs were slightly less from neat Transcutol than from water (ER = 0.88) potentially due to the dehydrating effect of neat Transcutol. One might speculate that the infinite dose of 133 mg donor solution/cm^2^ of skin may be less capable of dehydrating the skin over 24 h than the 1752 or 4688 mg/cm^2^ of neat Transcutol listed in Table IV.

Bayldon (5) investigated the effects of vehicles on the retention and permeation of lidocaine free base after application to sheep skin. The purpose of this study was to determine feasibility of a topical anesthetic veterinary product for use prior to minor surgical procedures. 88.5 μl/cm^2^ of lidocaine free base saturated solutions were dosed using Franz cells maintained at 32°C with PBS as the receptor solution. The authors emphasize that the clipped felt of sheep skin (the tissue loaded in the Franz cell for this study) had a continuous coat of an emulsion consisting of sweat and sebum. Sheep skin has dramatically more follicles than human skin, with Merino sheep having up to 10,000 follicles per cm^2^. The study found that non-aqueous (neat) vehicles (100% DMSO, 100% Transcutol, 100% IPM) enhanced penetration of lidocaine free base compared to the aqueous vehicles (0.5% or 1% sodium lauryl sulphate, 50% dimethyl sulfoxide, 50% Transcutol, or 100% water). A saturated solution dose of approximately 88.5 mg/cm^2^ is considered an infinite dose based upon FDA’s guidance that a finite IVPT dose should be 3–5 mg/cm^2^ (49). However, this quantity of Transcutol applied to sheep felt that has been processed to assure retention of a layer of sebum/sweat emulsion appears incapable of dehydrating the stratum corneum during IVPT. Thus, for topical application to sheep, the use of neat Transcutol in a clinically relevant dose of 88.5 μl/cm^2^ will enhance the skin transport of lidocaine.

In an IVPT study published by Gwak (45), the authors emphasized that the permeation fluxes of tenoxicam from saturated solutions in various vehicles were generally low (0.1–1.1 μg/cm^2^ h) and no individual vehicle possessed the necessary intrinsic activity to dramatically promote permeation. Upon closer examination of the reported permeation rates for water (0.15 ± 0.04 μg tenoxicam/cm^2^ h) and neat Transcutol (1.13 ± 0.42 μg tenoxicam/cm^2^ h), it is evident that the latter produced 7.5 times more tenoxicam compared to the saturated water (Table IV). This high ER for tenoxicam delivered from Transcutol could be explained by the inherently high variability in flux measurements of poorly permeating actives, or it could be an example of enhancement due to the solvent effect overwhelming the increased barrier resulting from neat Transcutol dehydrating the stratum corneum. Tenoxicam (45) has the most complex acid-base properties of the drugs considered in this review. At pH values below the pK_a1_ (4.3), tenoxicam carries a positive charge. At pH values above pK_a2_ (5.3), tenoxicam carries a negative charge and approaches 5 mg/ml solubility at the physiological pH of the viable epidermis. Based on this acid-base chemistry, neutral tenoxicam dissolved in Transcutol at 4.7 mg/ml should readily partition into the pH = 5 stratum corneum and just as readily partition out of the stratum corneum into the viable epidermis.

A consistent mechanistic picture of how during IVPT experiments neat Transcutol modifies the stratum corneum can be derived from the publications reviewed. If a true infinite dose (in excess of 1 ml/cm^2^ skin surface area) of neat Transcutol is added to the donor compartment for IVPT, then the stratum corneum dehydrates, stiffening the intercellular lipids and increasing the barrier of the skin. Simultaneously, Transcutol quickly permeates the stratum corneum potentially decreasing the barrier of the skin by decreasing drug charge (solvent effect), increasing the solubility of the active in the stratum corneum, and/or disorganizing the crystalline epidermal lipid structure. Based on the data summarized in Table IV, it is highly likely that the dehydration effect of dosing with neat Transcutol may dominate the competing penetration enhancing effects of dosing with neat Transcutol. This results in saturated water having higher skin flux values than saturated Transcutol.

It should be noted that the decrease in skin flux seen in these publications from neat Transcutol (Table IV) is an infinite dose IVPT artifact rather than a significant insight for developing a commercial topical product. In practice, when applied *in vivo*, 1 ml of a topical product will spread over 300 cm^2^ of skin surface area. This thin film of Transcutol will be present on the skin’s surface for less than a minute and will not be able to dehydrate the entire thickness of the stratum corneum. Thus, this mechanistic insight should be used to facilitate interpretation of steady-state IVPT data rather than guide formulation development.

### Effect of Transcutol Binary Solvent Blends on Drug Penetration/Permeation

With this understanding of how Transcutol partitions into the lipid filled intercellular spaces of the stratum corneum to retain the high skin permeability of a hydrated stratum corneum at reduced hydration conditions, it becomes apparent that blending Transcutol with molecules having complimentary mechanisms for disrupting epidermal lipid structure could result in synergistic skin penetration enhancement. This combined with Transcutol potentially reducing the charge of certain actives and increasing solubility and/or partition of some drugs into the stratum corneum results in optimized Transcutol blends consistently obtaining the highest enhancement ratios based on IVPT. Studies combining Transcutol with MCT, PG, PGML, PGMC, OA, IPM, propylene glycol laurate (PGL), or sucrose esters including sucrose myristate (SM), sucrose oleate (SO), and sucrose laurate (SL) have shown that Transcutol blends can dramatically increase the skin flux of certain pharmaceutical active ingredients (21,25,41,45,46,50–52). Skin flux enhancement ratios of over 50 were obtained for melatonin delivered from a TRC/PGL blend compared to skin flux from neat Transcutol (25). This ability of certain Transcutol blends synergistically enhancing drug penetration rates (for certain actives) is contrasted with the effect of lipophilic enhancer blends with Transcutol such as TRC/MCT for delivery of ivermectin (21) or TRC/IPM combinations studied for UV absorbers (33).

**Fig. 8:**
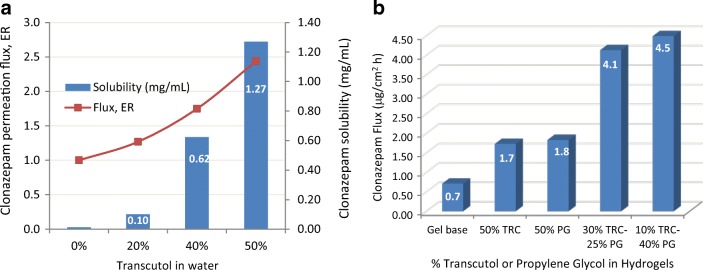
**a** Clonazepam flux enhancement ratio from different Transcutol concentrations in water. **b** Effect of Transcutol, propylene glycol, and their combinations on the flux of clonazepam across rabbit ear skin. Mura (53)

The influence of TRC, W, and PG combinations on clonazepam permeation through artificial membrane and excised rabbit ear skin from Carbopol hydrogels was characterized by Mura et al. (53). Franz cells loaded with rabbit ear skin were dosed at 312.5 mg gel/cm^2^ over a receptor solution of pH 7.5 PBS containing 25% *v*/*v* of PEG 400. The study concluded that Transcutol is a good enhancing carrier for clonazepam, yielding an ER = 3.4 (artificial membranes) or ER = 2.3 (rabbit ear skin) for flux rate as compared with that of the gel base (Fig. 8a). Moreover, when Transcutol was used in combination with PG (10% TRC:50% PG), a further increase in the flux rate was found, up to 6.3 times greater than gel base for the formulation (Fig. 8b). The authors attributed the enhancement of drug permeation to the solubilizing properties of Transcutol combined with its ability to increase drug cutaneous retention and to the penetration and carrier properties of PG across the skin.

**Fig. 9:**
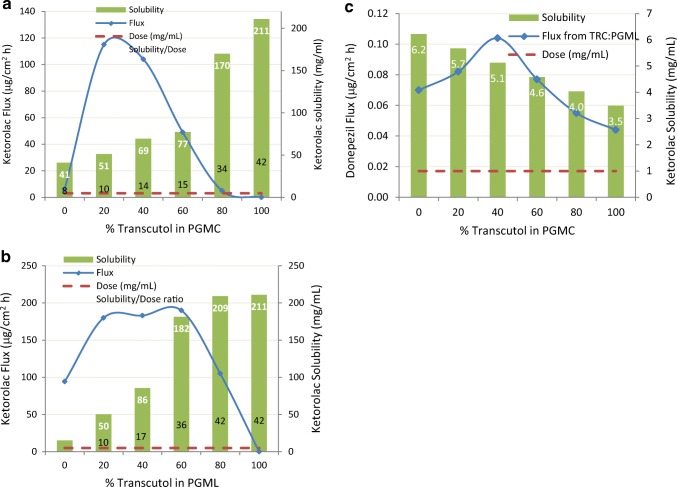
**a**–**c** Effect of Transcutol/propylene glycol monocaprylate (PGMC) or Transcutol/propylene glycol monolaurate (PGML) ratios on the permeation of ketorolac and donepezil from saturated solutions across excised hairless mouse skin. Adapted from Cho (41) and Kim (54)

The effect of TRC/PGMC and TRC/PGML blends were studied using infinite dosing of excised dorsal skin from hairless mice (6–8 weeks old) for ketorolac tromethamine (41), tenoxicam (45), ondansetron HCl (46), and donepezil HCl (54). Results for ketorolac (dosed at 5 mg/ml as the tromethamine salt) and donepezil (dosed at 1 mg/ml) are shown in Fig. 9. Since these studies did not dose at equivalent thermodynamic driving force, the skin flux values (blue solid lines) should be greatest when dosing (dashed red lines) are nearest drug saturation solubility (green bars) in Fig. 9a–c. This is clearly not the case for ketorolac or donepezil. The greatest skin penetration enhancement for donepezil occurs at 40% TRC and falls between 20 and 60% TRC for ketorolac. For both actives, skin flux is a compromise between the maximal flux caused by the synergistic ratio of TRC/PGMC or TRC/PGML and the loss of thermodynamic driving force for blends with significantly increased drug solubility and a drastic loss of permeation at elevated/neat Transcutol concentrations. It should be noted that more ketorolac was delivered from TRC/PGML mixtures than from the TRC/PGMC blends.

In another IVPT study of ketorolac tromethamine in a pressure sensitive transdermal delivery (Duro-Tak 87-2196R patch) system, Choi (50) tested 38 different combinations of various solvents/enhancers involving PG, MeOH, TRC, PGMC, and fatty acids. Much like the other reports, the combinations consisting of 40:60 ratio of TRC/PGMC or TRC/PGML showed the highest *in vitro* permeation profiles, in the Sprague-Dawley rat skin model. These formulations were preselected for pharmacokinetic (rat *in vivo*) experiments that followed and were compared with an orally administered dose. The results confirmed a comparative decrease in Cmax and prolonged Tmax and half-life for the ketorolac in transdermal systems, indicating a prolonged effect with reduced toxicity. Additionally, an excellent relationship was found between *in vitro* permeation flux and *in vivo* AUC (systemic delivery).

**Fig. 10:**
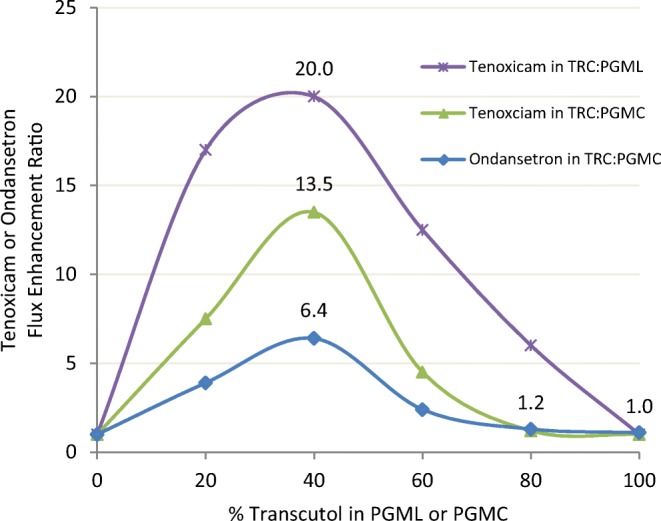
Effect of neat propylene glycol monocaprylate (PGMC), neat propylene glycol monolaurate (PGML), and binary mixtures of Transcutol with PGMC or PGML on drug permeation across excised hairless mouse skin. Enhancement ratios relate to data for saturated solutions of tenoxicam (45) and ondansetron (46)

Figure 10 presents the permeation enhancement (flux ER) data for similar TRC/PGMC and TRC/PGML blends saturated with either tenoxicam (45) or ondansetron HCl (46). Enhancement ratio was calculated as drug flux (μg/cm^2^ h) from the formulation divided by its flux from 100% TRC. Overall, the TRC/PG fatty ester ratio of 40:60 provided the maximum skin flux for both actives. More tenoxicam was delivered from TRC/PGML (ER = 20) than from TRC/PGMC (ER = 13.5). Tenoxicam was soluble up to 4.74 mg/ml in Transcutol, 1.27 mg/ml in PGMC, and 0.59 mg/ml in PGML. As for ondansetron, the highest ER ratio of 6.4 was obtained. Ondansetron solubility was higher in the TRC/PGMC blends, ranging from 18.1 mg/ml Transcutol to 1.0 mg/ml PGMC.

Since a decreased lag time occurred for the maximum ondansetron flux (ER = 6.4) a mechanistic narrative can be proposed. Transcutol disrupts structure in the region of the ceramide head group which allows the PG fatty ester greater penetration into the bilayer structured epidermal lipids. TRC/PGML blends appear to enhance penetration better than TRC/PGMC blends. Speculation concerning how and where the PG fatty ester disorders the structured epidermal lipids cannot be made based solely on IVPT results.

**Fig. 11:**
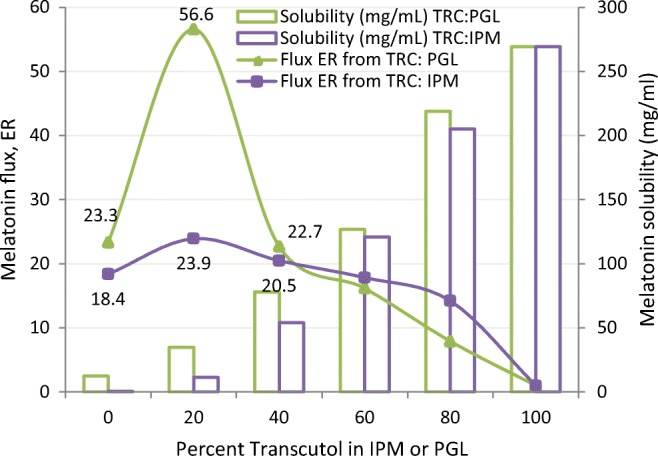
Effect of binary combinations of Transcutol with isopropyl myristate (IPM) or propylene glycol laurate (PGL) on excised hairless mouse skin permeation of melatonin. Gwak (25)

Binary mixtures of TRC/PGL and TRC/IPM were studied for permeation of melatonin across hairless mouse skin by Gwak (25). Solubility of melatonin increased with increasing ratio of Transcutol in the PGL or IPM. Flux enhancement ratios however varied significantly, the lowest observed for neat Transcutol and the highest synergistic effect with the 20:80 mixture of TRC/PGL (Fig. 11). It is worth noting that the experiments were run in triplicate and the largest standard deviation of the data set pertained to the 20:80 TRC/PGL mixture. Thus, the enhancement ratio of 56.6 (21.5 ± 7.34 μg/cm^2^ h) should not be viewed as rigorously quantitative, but the observation that TRC/PGL blends result in synergistic skin penetration enhancement for certain pharmaceutical active ingredients is consistent in the scientific literature. In contrast, when Transcutol was blended with IPM (25), melatonin skin permeation increased by a factor of 23.9 (at 20:80 ratio of TRC/IPM), dropping gradually at higher Transcutol ratios.

**Fig. 12:**
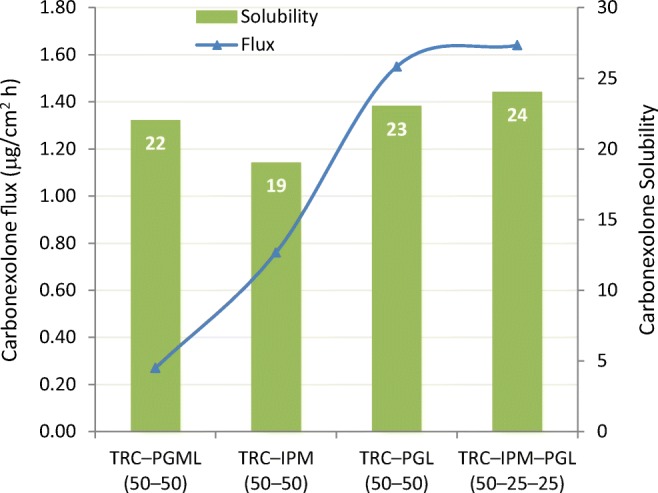
Solubility and flux of carbenoxolone from various mixtures of Transcutol with propylene glycol monolaurate(PGML), propylene glycol laurate (PGL), and isopropyl myristate (IPM). Adapted from Hirata (51)

**Fig. 13:**
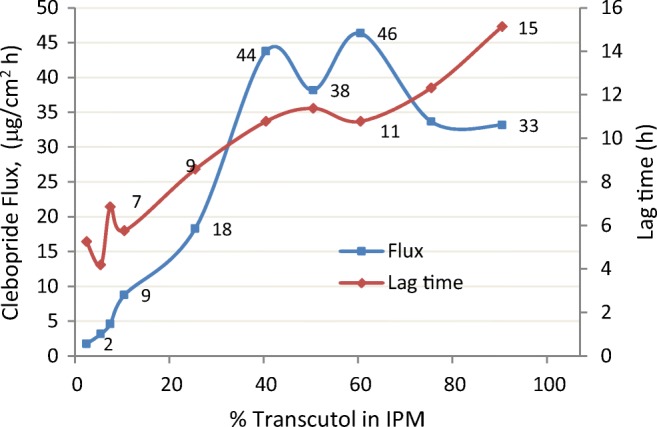
Lag time and flux of clebopride across rat skin from Transcutol and isopropyl myristate binary mixtures. Adapted from Rhee (55)

When saturated carbenoxolone solutions were dosed (Franz cell, 885 mg/cm^2^) on heat separated human skin (51), the 50:50 TRC/PGL solvent blend produced a drug flux of 1.55 μg/cm^2^ h compared to 0.76 μg carbenoxolone/cm^2^ h skin flux for a 50:50 TRC/IPM (Fig. 12). The highest carbenoxolone skin flux obtained in this study was 1.64 μg/cm^2^ h which required the blend of three excipients TRC/PGL/IPM (50:25:25). This additional example of Transcutol combined with IPM not synergistically enhancing skin penetration can be contrasted with results of another study involving clebopride (55) where increasing concentration of Transcutol in the TRC/IPM binary blends resulted in increased drug flux across the rat skin. As shown in Fig. 13, flux rates as high as 44 and 46 μg/cm^2^ h were obtained, representing 80- and 90-fold enhancement ratios respectively compared to that obtained from a 100% IPM control formulation (0.58 μg/cm^2^ h). Fig. 13 also shows the lag times observed for each of the binary mixtures studied, increasing from 5 h (100% IPM formulation) to 15 h from the 100% Transcutol formulation. A 15-h lag time in a 24-h infinite dose IVPT indicates that lipids are being extracted from the skin into the donor phase to cause a sudden loss of the skin barrier. Thus, the 80-fold increase in clebopride skin penetration for a 40:60 blend of TRC/IPM compared to IPM alone (as control) must be viewed in terms of the experimental design.

**Fig. 14:**
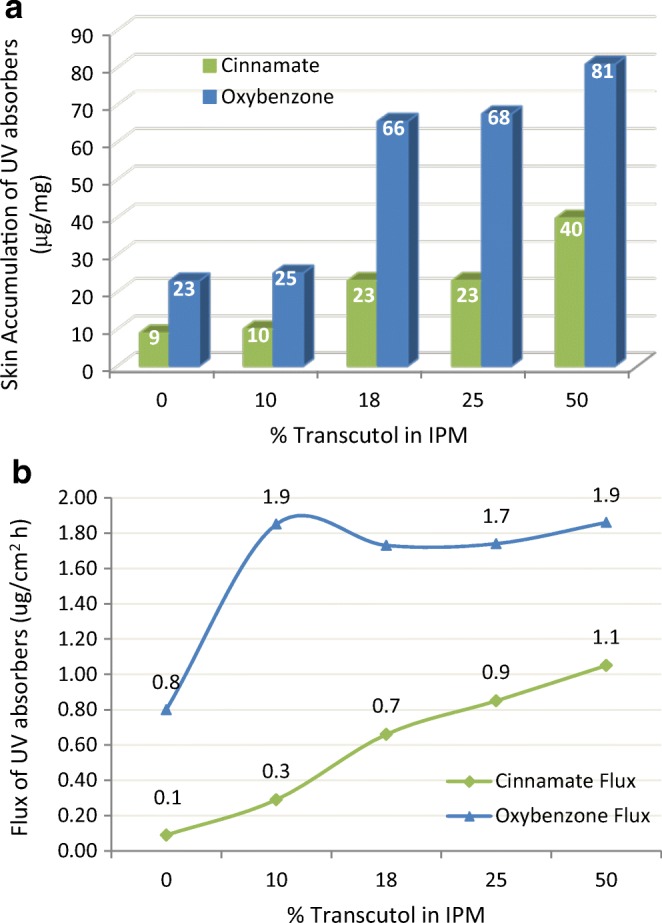
**a** UV absorber concentration in the skin (accumulation). **b** UV absorber permeation rate from various combinations of Transcutol and isopropyl myristate. Adapted from Godwin (33)

Godwin (33) examined the effect of TRC/IPM mixtures on the skin accumulation as well as skin permeation of UV absorbers oxybenzone (log *P* = 3.8) and octyl-4-methoxy cinnamate (cinnamate, log *P* = 5.8). These experiments used Franz cells loaded with hairless mouse skin, dosed at 312.5 mg/cm^2^ (6% *w*/*w*) UV absorber dissolved in IPM or TRC/IPM blends. Per the results summarized in Fig. 14a, the increases in skin accumulation were statistically significant for both UV absorbers at Transcutol concentrations of 18, 25, and 50%, with oxybenzone having twice the skin accumulation compared to cinnamate. The flux values (Fig. 14b) were also higher for oxybenzone than with cinnamate and this without statistical significance. The researchers concluded that inclusion of Transcutol led to increased skin accumulation of oxybenzone and cinnamate without a concomitant increase in transdermal permeation.

**Fig. 15:**
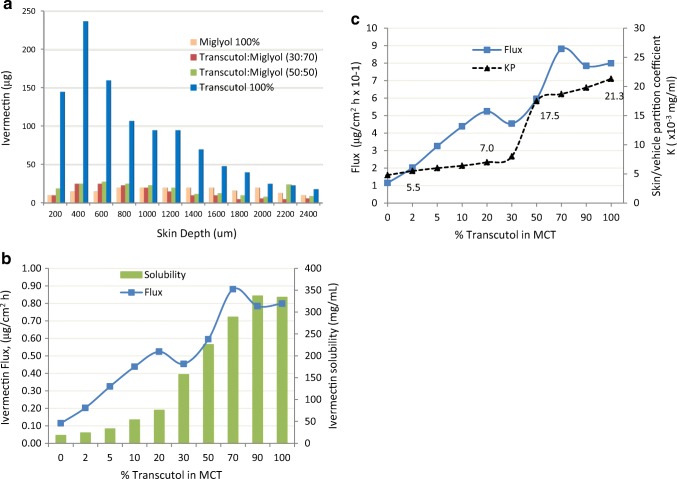
**a** The distribution of ivermectin in bovine skin after topical application of ivermectin solutions (14 mg/ml) for 66 h *in vitro*. **b** Effect of solvent composition on the solubility and permeation rate of ivermectin across bovine skin. **c** Partition coefficient of ivermectin solutions (14 mg/ml) in Transcutol and MCT (medium-chain triglyceride) binary mixtures. Adapted from Yazdanian (21)

The effect of Transcutol concentration on active flux was evident in the work of Yazdanian and yet certain conclusions drawn from that work need closer examination. In Yazdanian (21), the authors were developing a veterinary topical ivermectin product for cattle. As part of the study they prepared ivermectin solutions (14 mg/ml) in Miglyol 812 (MCT) and Transcutol blends of 90/10, 70/30, 50/50, and 0/100 (neat Transcutol). They traced tritiated ivermectin as it crossed excised bovine skin (Hostein, dermatomed to 4.5 ± 0.2 mm) and measured the appearance of Transcutol in the receptor solution (gas chromatography). The distribution profiles of ivermectin as a function of skin depth and solvent composition are summarized in Fig. 15a. As shown, the amount of ivermectin present in the skin was significantly greater, with most of the ivermectin localized in the upper layers of the skin. The authors reported that ivermectin permeation was accompanied by the formation of cutaneous depots of ivermectin; that partitioning of ivermectin into the skin was far greater in the presence of Transcutol in saturated solutions; that ivermectin “was permeating with Transcutol, in which it is very soluble.” It was also noted that there were no significant statistical differences in the flux of Transcutol as the concentration of Transcutol in the donor phase was increased. This was in contrast to what was expected on the basis of thermodynamic activity. A closer examination of the data (Fig. 15b, c), however, reveals that overall, ivermectin solubility as well as skin permeation parameters (flux and Kp) increased significantly with the increasing concentration of Transcutol. Since this IVPT study (21) was completed in support of a veterinary product, it is important to note that the Franz cell study (dosed at 282 mg/cm^2^ using a receptor solution of 25% glycerol in water) held the skin temperature at 39°C to match the skin temperature of living cattle. No quantitative correlation between bovine and human skin transport should be inferred from this study; however, the observation of how flux and permeability coefficient trend with increasing Transcutol concentration is noteworthy.

**Fig. 16:**
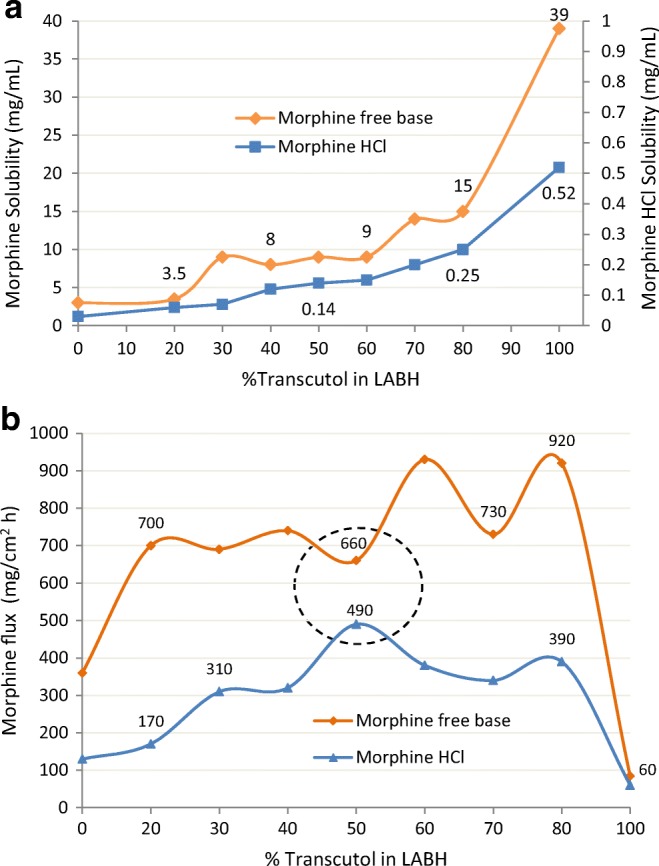
**a** Morphine solubility in binary blends of Transcutol and Labrafac (LABH). **b** Morphine flux from binary blends of Transcutol and LABH. Adapted from Rieg-Falson (56)

Rieg-Falson (56) blended Transcutol with Labrafac Hydro, a liquid non-ionic surfactant consisting of caprylic/capric triglyceride PEG-4 esters (LABH). The liquid mixtures of TRC/LABH were saturated with radiolabeled morphine free base or radiolabeled morphine HCl and then these formulations were gelled with 10% Ethocel 20. They mounted hairless mouse abdominal skin on a diffusion cell, dosed at 394 mg/cm^2^, and followed skin permeation for 96 h. In general, solubility for both the free base and hydrochloride salt of morphine increased with increasing Transcutol concentrations, with morphine HCl being about 60-fold less soluble in the formulations than the free base (Fig. 16a). The morphine flux values (56) were very similar to those reported by Yazdanian (21) for ivermectin. As shown (Fig. 16b), skin flux increased with increasing concentrations of Transcutol until plateauing at ~ 30% Transcutol. The flux of both actives from neat Transcutol dramatically decreased, likely due to neat Transcutol dehydrating the stratum corneum and increasing the barrier properties of the skin. Since all of the formulations were at equivalent, maximum thermodynamic driving force, the marked difference in the flux values between the morphine free base and the morphine HCL may be attributed to the lower permeability of the latter, *i.e.*, the HCL salt being in ionized form. Noteworthy is the proximity of the nearly equal flux values at 50:50 TRC/LABH for both species of the morphine, indicating the possibility of a solvent effect being responsible for reducing the morphine HCl charge.

In a human volunteer study (39) MCT gelled with 7.5% polypropylene was the control formulation for delivering methyl nicotinate. Increased blood flow induced by this active that rapidly crosses the stratum corneum was measured using laser Doppler flowmetry. 0.25 ml of the gelled, non-aqueous test articles were applied to the forearm of volunteers using a Hilltop (occlusive) chamber. Addition of 10% Transcutol resulted in a statistically significant enhancement factor of 4.6 ± 2.7 for 10% Transcutol formulation compared to the control formulation at 0.5-h application time. It should be noted that the relative bioavailability enhancement factor calculated by these researchers is very different than the simple ER values used throughout this review. The researchers also studied the test article application sites using ATR-FTIR spectroscopy. The ATR-FTIR band intensity indicated that the MCT/TRC blend appeared to retain more water in the stratum corneum compared to control, but that neither significant disorder of the bilayer structured intercellular lipids nor significant protein configuration changes occurred when Transcutol was added.

**Fig. 17:**
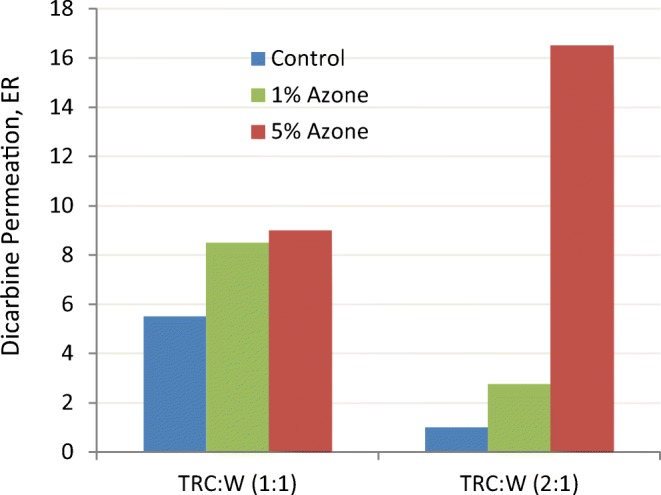
Permeation enhancement ratios for dicarbine (stobadine HCl) across rat skin from Transcutol/water combinations with 1% and 5% azone. From Koprda (18)

Interestingly, the relative optimization of drug flux at 50:50 blends with Transcutol is a recurring theme in a number of other studies (56–58). Koprda (18) studied permeation in the rat skin of dicarbine (stobadine HCl) from TRC/W solutions at 1:1 and 2:1 ratio, with and without azone. The results summarized in Fig. 17 show that drug permeation increased with the addition of 1% and 5% azone and that the drug permeation was effectively higher from the 50:50 (TRC/W) than with the 66:33 (TRC/W) mixture, even if 1% azone was added. However, addition of 5% azone to the 2:1 TRC/W combination increased the flux dramatically by 16 times. The enhancing effect of azone has been attributed to direct interactions with the stratum corneum lipids and lowering their melt transition temperature, thus creating a more fluid environment amenable to permeant diffusion. The ability of Transcutol to preferentially partition into more polar environments and disturb the packing in the interfacial head group layer of the intercellular lipids works synergistically with azone’s ability to melt the natural orthorhombic structure of the hydrocarbon chain packing of the intercellular lipids. This synergism between Transcutol and azone appears to be greatest at a specific ratio of Transcutol, water and azone. In addition to Transcutol facilitating azone fluidizing of the intercellular lipids, Transcutol is likely enhancing the solubility of dicarbine in the skin (22,28,43).

**Fig. 18:**
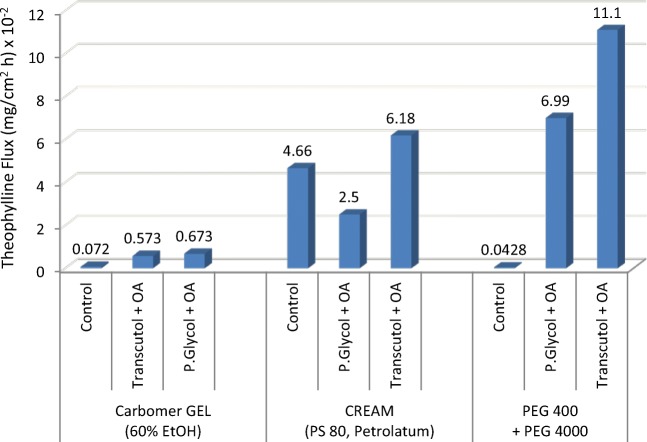
Flux of theophylline from various bases containing PG/OA or TRC/OA combinations. Adapted from Touitou (57)

Addition of OA to TRC can produce an additive enhancing effect on the delivery of water soluble actives like theophylline (57) and caffeine (58). Excised hairless mouse skin mounted on side-by-side diffusion was used for both actives. In the theophylline study, Touitou (57) added a blend of 20% TRC and 10% OA to three bases, namely a carbomer gel consisting of 60% EtOH, a petrolatum and polysorbate 80 (PS 80)-based cream, and a hydrophilic PEG 400/PEG 4000 ointment base. The concentration of the tritiated theophylline in the formulation was at 10 mg/g. Fig. 18 summarizes the permeation rate of theophylline from each of these formulation bases where the TRC/OA/PEG base (2:1:7) combination produced the highest flux (11.1 mg/cm^2^ h) representing an enhancement ratio of 260 *vs.* control (0.0428 mg/cm^2^ h) and flux ER of 1.6 against the PG/OA/PEG base (6.99 mg/cm^2^ h) combination.

**Fig. 19:**
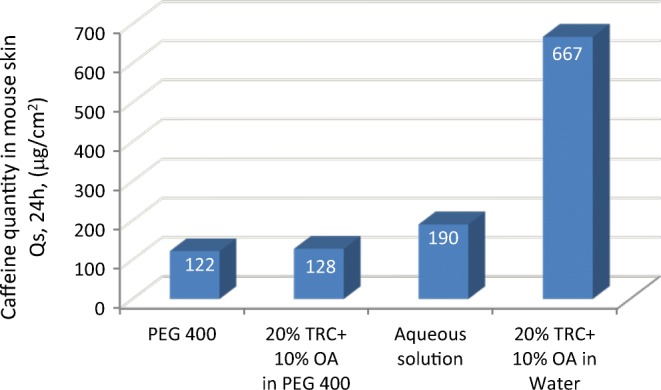
Effect of formulations on the flux of caffeine through hairless mouse skin. Adapted from Touitou (58)

In a later study (58), this same skin penetration enhancing 2:1:7 combination of TRC/OA/PEG base was tested for delivery of 3% caffeine. The findings for caffeine were similar in that the best additive combination effect was associated with the TRC/OA blend as enhancer. This latter study however took additional steps to examine the effect of hydration (water in the formulation) (Fig. 19) with results that highlighted the importance of water in the enhancement or formulation optimization.

It should be noted that OA, especially at high concentrations, can disrupt the lipid domains within the stratum corneum by forming pools in the intercellular space to create “pores.” These pores provide less resistance for polar molecules resulting in skin penetration enhancement. Non-polar molecules have also been shown to be enhanced when OA pools in the intercellular space of the lipid domain. The original research characterizing this phenomenon was concisely reviewed by Benson (23) .

**Fig. 20:**
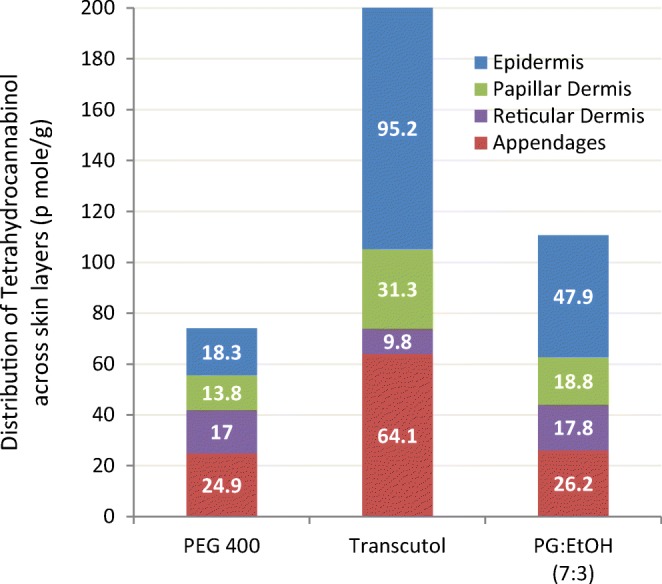
Distribution of THC in the various skin layers of rat skin delivered within 24 h by three carrier systems. Adapted from Fabin (59)

This next example involving the highly lipophilic tetrahydrocannabinol (THC) helps compare the penetration properties of Transcutol against OA in different carrier systems. To assess whether drug carriers could influence the drug pathway through the skin, Fabin (59) incorporated THC or OA as permeant into three enhancer systems: neat TRC, PEG 400, and a 7:3 blend of PG/EtOH. Formulations with 0.8 g THC (50 μCi drug) were applied to 3.2 cm^2^ surface area of rat skin *in vivo.* Samples of the skin were then analyzed *ex vivo* at 2 and 24 h after application, using quantitative autoradiography to visualize and measure the trace levels of tritiated THC penetrating the different layers of the skin. The highest penetration for THC was observed with the Transcutol formulation after 2 h of application, with no significant difference between the THC and OA concentrations accumulating in the skin layers. Increasing the study duration to 24 h however helped emphasize the preference of THC or OA to choose the penetration route, demonstrated by a much greater skin penetration, as well as a different localization for both but THC having a significantly higher overall distribution than OA. The penetration results from the three enhancer systems for THC are summarized in Fig. 20, showing that the concentrations delivered from neat Transcutol were significantly higher compared to PEG 400 or the PG/EtOH mixture. Comparing the 24 h data to those obtained at 2 h of application, the authors noted that the skin distribution profile of THC and OA had changed over time with the PG/EtOH (7:3) formulation, whereas it was unchanged from the neat Transcutol formulation. This indicated that Transcutol, unlike OA, does not change the skin barrier/permeability over time.

### Drug Transport from Transcutol and Sucrose Ester Mixtures

**Fig. 21:**
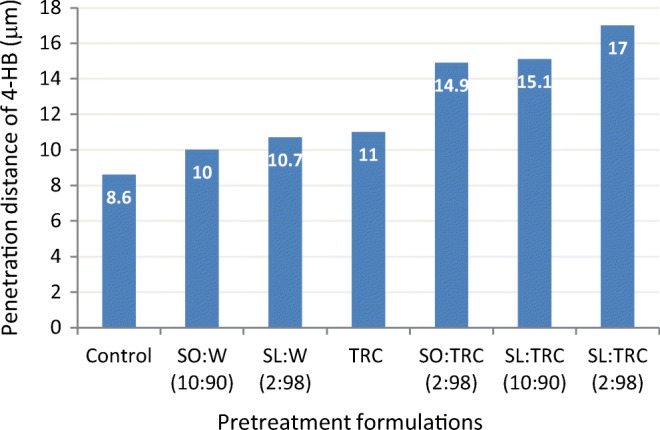
Distribution of 4-HB in healthy human skin (*in vivo*) measured by FTIR. Adapted from Ayala Bravo (60)

In a study involving healthy human volunteers, Ayala Bravo (60) used FTIR and TEWL measurements to assess the effect of TRC, SO, SL, and their combinations on the penetration depth of a model drug, 4-hydroxybenzonitrile (4-HB). The model penetrant was selected on the basis of its intense C≡N stretching absorbance at 2230 cm^−1^, permitting it to be monitored by ATR-FTIR spectroscopy. Drug penetration distances were measured after a pretreatment (1-h application) with various TRC/sucrose ester combinations. Whereas the distribution of 4-HB for the control formulation suggested that the active is able to distribute itself in the stratum corneum independent of enhancers, the extent of its penetration was clearly influenced by the addition of the enhancer/combinations (Fig. 21). The penetration distance of 4-HB from neat Transcutol was similar to the aqueous solutions of sucrose esters, SO/W (10:90) and SL/W (2:98). Referring to a prior study where no modification of stratum corneum lipids had been observed for sucrose ester aqueous solutions, the authors pointed to a distinct lipid fluidization in the presence of SL-TRC and suggested that the SL absorption in the stratum corneum was facilitated by TRC.

**Fig. 22:**
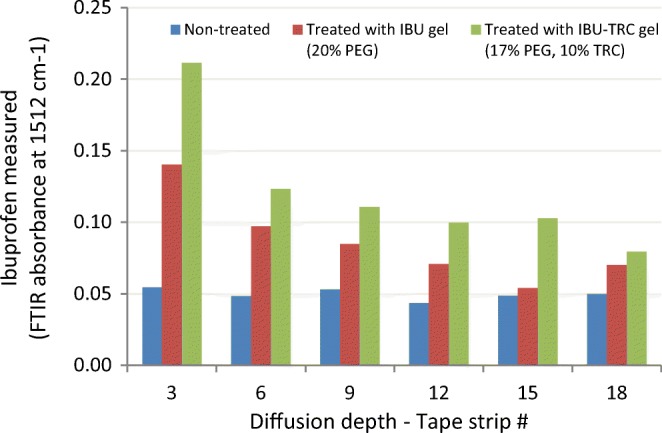
Ibuprofen levels in the stripped human skin layers measured by FTIR. Adapted from Csizmazia (61)

In developing ibuprofen transdermal gel formulations, Csizmazia (61) dissolved 5% ibuprofen in PEG 400 (20%), followed by adding the solution to Carbopol 971 hydrogel to serve as control formulation. Similarly, hydrogels consisting of 2.64% SL or 10% TRC were prepared and tested on excised human skin. Per the IVPT results, the skin permeation of ibuprofen increased from the SL formulation by over 2-fold, whereas the permeation from the neat TRC formulation was about half that from the control formulation. In the same study, the FTIR analysis of 3 to 18 strips taken from the treated skin samples confirmed a higher amount of ibuprofen for the Transcutol formulation compared to the control (Fig. 22). The authors did not elaborate if the three formulations had equivalent solubility/thermodynamic forces. However, the study concluded that Transcutol is an effective diffusion enhancer while the sucrose ester is a permeation promoter for ibuprofen.

Ibuprofen penetration and permeation studies by Balazs (62) and Cazares-Delgadillo (63) also involved combinations of Transcutol with sucrose esters. Balazs (62) prepared formulations PEG 400 aqueous carbomer gels consisting 10% TRC and 2.64% SL or SM mixtures. Heat-separated human epidermis was loaded in a Franz cell and dosed at 170 mg/cm^2^ with a dissolved 5% ibuprofen PEG 400 aqueous carbomer gel. The gel consisting of TRC/SL produced an enhancement ratio of 0.80 for skin flux and 0.95 for skin concentration against the gel without enhancer. This compares to the more effective blend of TRC/SM which had an enhancement ratio of 1.7 for skin flux and 1.7 for skin concentration.

The effect of SL or SO combined with TRC on the percutaneous penetration of lidocaine as a function of ionization was determined by Cazares-Delgadillo (63). In this study pig ear skin was loaded in a Franz cell (0.8 cm^2^) and pretreated for 1 h with 100 μl of the formulation, *i.e.*, 2% SL in TRC or 2% SO in TRC. The pretreatment solution was removed with a cotton swab followed by application of 300 μl of a saturated lidocaine hydrochloride solution in phosphate buffer at pH 5.0 (unionized fraction = 0.00), pH 7.0 (unionized fraction = 0.11), and pH 9.0 (unionized fraction = 0.93). The diffusion of lidocaine was significantly improved when the skin was pretreated with both TRC/SL and the TRC/SO (98:2) formulations. However, the effect of each ester on the penetration of the ionized and unionized forms was quite distinct: SL favored the diffusion of the ionized species (pH 5.0 ER = 11.9 and pH 7.0 ER = 10.8) but reduced the passage of the uncharged base (pH 9.0 ER = 0.6). In contrast, SO enhanced the permeation of both the ionized and unionized species of lidocaine (pH 5.0 ER = 3.8, pH 7.0 ER = 3.4, and pH 9.0 ER = 2.7).

### Simple Gel Formulations with 1–50% Transcutol

For the 12 pharmaceutical actives listed in Table V (enhancement of skin flux) and Table VI (enhancement of skin retention), adding 1–50% Transcutol to a simple gel formulation resulted in ER values of 1–4.8 for skin flux and ER values of up to 3.4 for skin retention. For the studies that varied the amount of Transcutol in the formulation, the ER generally increased with increasing amounts of Transcutol. Addition of up to 50% Transcutol to a simple gel formulation resulted in enhanced skin penetration, presumably by partitioning into the skin and allowing the epidermal lipid domain to retain the high skin permeability of a hydrated stratum corneum and depending on the active potentially decreasing drug charge (solvent effect) or increasing solubility/partitioning of the active in the stratum corneum.

**Table V:** Effect on Permeation Rate: Compilation of How Addition of 1–50% Transcutol to a Topical Formulation Influences Skin Flux of a Pharmaceutical Active Ingredient

Reference	Skin type	Receptor	Dose (mg/cm^2^)	Duration (h)	% API	Active	% TRC	Flux (μg/cm^2^ h)	ER^*a*^
2000 Mura (53)	Rabbit ear	PBS/PEG 400	312	16	1.50	Clonazepam	50	1.73	2.4
							40	1.24	1.7
							20	0.9	1.3
2004 Gungor (64)	Rat skin	PBS/EtOH 75:25	318	24	1	Nimesulide	40	14.1	1.8
2011 Chadha (13)	Human	PBS/EtOH 80:20	312	24	0.50	Genistein	25	1.2	4.8
2001 Puglia (32)	Human-SCE	50:50 W/EtOH	133	36	1	Lorazepam	20	0.97	2.0
						Clonazepam		0.55	1.7
2010 Barakat (65)	Rat clipped	PBS/EtOH 70:30	588	12	5	Naproxen	1	146	1.8
							2	250	3.0
							4	94	1.1
2011 Javadzadeh (66)	Rat clipped	PBS	264	6	1	Methotrexate	2	56	3.4

^*a*^ER is calculated as drug flux from Transcutol formulation divided by the drug flux from the control formulation (without Transcutol) reported in the original publication

**Table VI:** Effect on Skin Retention: Compilation of How Addition of 5–20% Transcutol to a Topical Formulation Influences Skin Retention of a Pharmaceutical Active Ingredient

Reference	Skin	Receptor/study	Dose (mg/cm^2^)	Duration (h)	Test area	% API	Active	% TRC	Retention	ER^*a*^
2009 Senyigit (67)	Pig ear	PBS/EtOH (70:30)	Infinite	6	Epidermis	0.05	Clobetasol-17 propionate	20	0.3 μg/mg	3.4
					Dermis				0.004 μg/mg	0.9
					Epidermis	0.1	Mometasone furoate	20	1.2 μg/mg	1.4
					Dermis				0.019 μg/mg	1.7
2014 Berko (68)	Mouse	(*In vivo* animal study)	Infinite	6	Skin	5	Ibuprofen	10	189 μg/cm^2^	2.8
2014 Tiossi (69)	Pig ear	PBS (pH 7.2)	512	12	Stratum Corneum	~ 2	Solasonine	5	20 μg/cm^2^	1.1
							Solamargine		22 μg/cm^2^	1.2

^*a*^ ER is calculated as drug retention for Transcutol formulation divided by the drug retention for the control formulation (without Transcutol) reported in the original publication

### The Intracutaneous Depot

Starting in 1988, Ritschel and his group (1,19,34,35,40,67,70,71) published a series of papers showing that Transcutol in certain topical preparations could form an “intracutaneous depot”: for griseofulvin (19), coumarin (70), meperidine (71), papaverine (72), and clobetasol 17-propionate (67).

In the frequently cited 1991 publication (34), a 0.09% hydrocortisone gel consisting of 50% Transcutol, 12.00% PG, 10.00% Cab-O-Sil, and triethanolamine buffer pH 8.0 q.s. ad to 100% was prepared. Sprague-Dawley rats were dosed daily by applying 1 g of gel into a flexible Teflon ring (4.24 cm^2^). The dosing area was then closed with a polyethylene membrane and secured with bandage around the body of the rat. The skin under the dosing area was excised and prepared for autoradiography and morphological (both light and electron microscopy) study. Ritschel (34) proposed the following mechanism for forming the intracutaneous depot. “The major barrier for the absorption of drugs through the skin is the stratum corneum….The intercellular space volume is relatively small (1–10%) and may be a major pathway for permeation but at the same time the intercellular lipids are important in controlling the percutaneous absorption. The swelling of intercellular spaces, and the accumulation of foreign material outside the cell membrane is seen in the presence of Transcutol (evident from electron micrographs). It appears that Transcutol may incorporate into the multiple bilayer due to its polar and non-polar nature and thereby swell the intercellular lipids without altering the multiple bilayer structure. These swollen lipids may hold hydrocortisone and thereby form an intracutaneous depot in the presence of Transcutol, since hydrocortisone has high affinity for Transcutol.”

**Fig. 23:**
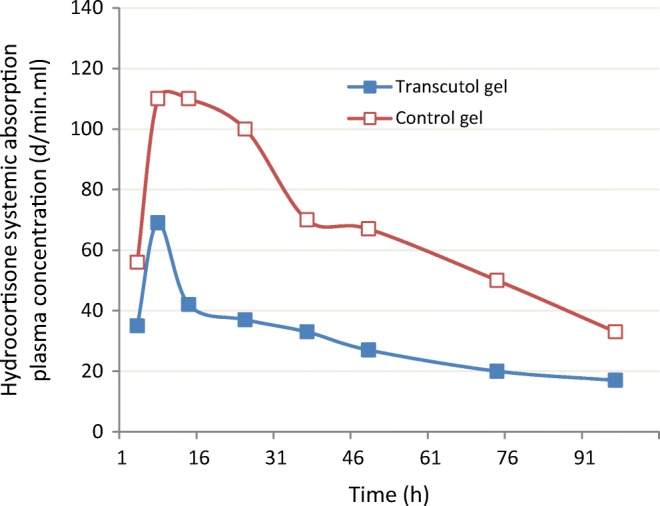
Amount of radioactivity present in 1 ml of whole blood over 96 h after single dose administration of hydrocortisone gel. From Panchagnula (40)

The aforementioned gel formulation (50% TRC:12% PG:10% Cab-O-Sil) was evaluated by Panchagnula (40) for delivery of hydrocortisone and dexamethasone *in vitro* and *in vivo*. The amount of steroid permeating across the rat skin (*in vitro*) was significantly less compared to the control formulation (without Transcutol). This was concurrent with a 3-fold increase in the skin concentration of hydrocortisone with the Transcutol formulation. The subsequent *in vivo* rat experiments involving radiolabeled hydrocortisone, obtained by measuring the total radioactivity in the blood for up to 96 h corroborated with the *in vitro* results. After a single dose administration, the amount of hydrocortisone measured in the plasma was 6.06 ± 1.27 d min^−1^ ml^−1^ h for the Transcutol formulation compared with 2.52 ± 0.43 × 106 d min^−1^ ml^−1^ h for the control formulation, indicating a 58% reduction in body burden (Fig. 23).

After review of the Transcutol literature in light of the work by Ritschel and colleagues, formation of the intracutaneous depot occurs for a subset of actives that after being dissolved in Transcutol have significantly greater solubility in the stratum corneum. These drugs also have low water solubility (such as clobetasol 17-propionate at 0.004 mg/ml, griseofulvin at 0.008 mg/ml), papaverine at 0.013 mg/ml, dexamethasone at 0.089 mg/ml, or hydrocortisone at 0.32 mg/ml) or large octanol-water partition coefficients (such as meperidine having a log *P* = 2.7, clobetasol 17-propionate having a log *P* = 3.5, or papaverine having a log *P* = 4.2) and will not readily partition out of the stratum corneum into the viable epidermis. For this subset of drugs, certain *in vitro* techniques result in remarkably high concentrations of active being measured in the skin. Thus, the intracutaneous depot could also be termed Transcutol-enhanced stratum corneum reservoir function (73) .

## CONCLUSION

This review was limited to a discussion of the enhancing mechanisms associated with simple Transcutol formulations, with a critical eye for the IVPT methodologies used in the published works. As presented, certain conclusions from past publications may be based on far less sophisticated evaluation techniques. An improved understanding of the skin physiology and emergence of new analytical techniques has facilitated this retrospective analysis of the past publications under a new light.

Clearly, the role of Transcutol as drug solubilizer and/or enhancer for skin penetration, permeation, or retention varies depending on its use concentration (neat or diluted) and its mixtures with other enhancers (formulation). Alongside enhanced permeability, an increase of drug concentration in the skin is commonly observed with Transcutol formulations. This effect must not be confounded with certain reports of drug depot effect for Transcutol which in retrospect appear to be more dependent on the physical properties of the active and artifacts inherent to infinite dosing IVPT, than a phenomenon unique to Transcutol. The unique feature of Transcutol that should be the focus of formulation scientists is that Transcutol can readily penetrate the stratum corneum and strongly interact with the water of the intercellular path. This Transcutol/water interaction can modify the stratum corneum barrier in several ways. As Transcutol permeates the stratum corneum, it modifies the molecular mobility of the stratum corneum protein and lipids to be similar to PBS soaked skin, *i.e.*, decreases skin barrier function. When applied at high concentrations or neat, Transcutol dehydrates the stratum corneum and increases the skin barrier function. The skin permeation of Transcutol leads to increased drug solubility in the stratum corneum, decreasing the skin barrier for actives that readily partition into the viable epidermis and increases stratum corneum retention for actives that do not readily partition into the viable epidermis. The permeation of Transcutol facilitates also the co-administration of other penetration enhancers known to disrupt (fluidize) epidermal lipid structure, *i.e.*, decrease in skin barrier.

Other important considerations highlighted in this review were monitoring of the changes in thermodynamic driving force and drug properties notably solubility and log *P* and charge. Important to consider is the potential solvent effect, *i.e.*, the ability of Transcutol to decrease charge of ionizable drugs due to its low dielectric constant when evaluating it as skin penetration enhancer.

Acronyms

**Table:** 

Acronym	Description	Trade name
DEGEE	Diethylene glycol monoethyl ether	Transcutol®
DEGME	Diethylene glycol methyl ether	–
EtOH	Ethanol	–
IPM	Isopropyl myristate	–
IVPT	*In vitro* permeation test	
LABH	Caprylo-caproyl 4-macrogolglycerides	Labrafac™ Hydrophyl
LABS	Caprylo-caproyl 6-macrogolglycerides	Labrasol®
MEOH	Methanol	–
MCT	Medium-chain triglycerides	Miglyol®, Labrafac™
OA	Oleic acid	–
PBS	Phosphate buffer solution	–
PEG	Polyethylene glycol	–
PG	Propylene glycol	–
PGL	Propylene glycol laurate	Lauroglycol™ FCC
PGMC	Propylene glycol monocaprylate	Capryol™ 90
PGML	Propylene glycol monolaurate	Lauroglycol™ 90
SE	Sucrose ester	
SL	Sucrose laurate	
SM	Sucrose myristate	
SO	Sucrose oleate	
TRC	Transcutol	–
W	Water	–
